# The *I*_K1_/Kir2.1 channel agonist zacopride prevents and cures acute ischemic arrhythmias in the rat

**DOI:** 10.1371/journal.pone.0177600

**Published:** 2017-05-18

**Authors:** Xu-Wen Zhai, Li Zhang, Yun-Fei Guo, Ying Yang, Dong-Ming Wang, Yan Zhang, Pan Li, Yi-Fan Niu, Qi-Long Feng, Bo-Wei Wu, Ji-Min Cao, Qing-Hua Liu

**Affiliations:** 1 Department of Physiology, Shanxi Medical University, Taiyuan, China; 2 Shanxi Provincial Childrens Hospital, Taiyuan, China; 3 Department of Pathophysiology, Shanxi Medical University, Taiyuan, China; 4 The Second Hospital, Shanxi Medical University, Taiyuan, China; 5 Department of Physiology, Institute of Basic Medical Sciences, Chinese Academy of Medical Sciences, School of Basic Medicine, Peking Union Medical College, Beijing, China; Max Delbrueck Center for Molecular Medicine, GERMANY

## Abstract

Arrhythmogenesis in acute myocardial infarction (MI) is associated with depolarization of resting membraine potential (RMP) and decrease of inward rectifier potassium current (*I*_K1_) in cardiomyocytes. However, clinical anti-arrhythmic agents that primarily act on RMP by enhancing the *I*_K1_ channel are not currently available. We hypothesized that zacopride, a selective and moderate agonist of the *I*_K1_/Kir2.1 channels, prevents and cures acute ischemic arrhythmias. To test this viewpoint, adult Sprague-Dawley (SD) rats were subjected to MI by ligating the left main coronary artery. The antiarrhythmic effects of zacopride (*i*.*v*. infusion) were observed in the settings of pre-treatment (zacopride given 3 min prior to coronary occlusion), post-treatment (zacopride given 3 min after coronary occlusion) and therapeutic treatment (zacopride given 30 s after the onset of the first sustained ventricular tachycardia (VT)/ventricular fibrillation (VF) post MI). In all the three treatment modes, zacopride (15 μg/kg) inhibited MI-induced ventricular tachyarrhythmias, as shown by significant decreases in the premature ventricular contraction (PVC) and the duration and incidence of VT or VF. In Langendorff perfused rat hearts, the antiarrhythmic effect of 1 μmol/L zacopride were reversed by 1 μmol/L BaCl_2_, a blocker of *I*_K1_ channel. Patch clamp results in freshly isolated rat ventricular myocytes indicated that zacopride activated the *I*_K1_ channel and thereby reversed hypoxia-induced RMP depolarization and action potential duration (APD) prolongation. In addition, zacopride (1 μmol/L) suppressed hypoxia- or isoproterenol- induced delayed afterdepolarizations (DADs). In Kir2.x transfected Chinese hamster ovary (CHO) cells, zacopride activated the Kir2.1 homomeric channel but not the Kir2.2 or Kir2.3 channels. These results support our hypothesis that moderately enhancing *I*_K1_/Kir2.1 currents as by zacopride rescues ischemia- and hypoxia- induced RMP depolarization, and thereby prevents and cures acute ischemic arrhythmias. This study brings a new viewpoint to antiarrhythmic theories and provides a promising target for the treatment of acute ischemic arrhythmias.

## Introduction

In the past three decades, elucidation of the structure and function of cardiac ion channels has contributed to our understanding of electrophysiological changes, arrhythmogenesis and the treatment of arrhythmias in cardiac diseases, including myocardial infarction (MI). Current clinical antiarrhythmic drugs primarily act on Na^+^, K^+^ and Ca^2+^ channels, and most of them are channel blockers. When producing therapeutic advantage against arrhythmias, these channel blockers may lead to unwanted electrophysiological abnormalities and proarrhythmic risks. Therefore, it is necessary to develop new antiarrhythmic agents based on new antiarrhythmic theory/mechanism. Cardiac inward rectifier potassium (Kir) channels constitute the *I*_K1_ channels which are present in all ventricular and atrial myocytes and are important for stabilizing the resting membrane potential (RMP). *I*_K1_ channels establish the excitation threshold and modulate the final repolarization phase of the action potential (AP) in cardiomyocytes, [1, 2] and thus exert profound effects on cardiac excitability and arrhythmogenesis.

Acute cardiac ischemia in coronary heart disease is a leading cause of sudden death primarily due to lethal arrhythmias, including ventricular tachycardia (VT), ventricular fibrillation (VF) and cardiac arrest [3]. In case of ischemia, diastolic potential decline [4–6] and catecholamine secretion [7–9] are two critical factors underlying the ventricular arrhythmias. Considering the key role of *I*_K1_ channel in maintaining the RMP, and the fact that *I*_K1_ is decreased by lysophosphatidylcholine (LPC) [6], intracellular acidosis [10, 11], and β-adrenergic receptor (β-AR) agonist [12], *I*_K1_ may probably be involved in the arrhythmogenesis in ischemic heart diseases. However, drugs that act mainly on RMP or *I*_K1_ channel are currently not available in the clinic. Zacopride is originally known as a 5-HT_3_ receptor antagonist and a 5-HT_4_ receptor agonist. In 2012 and 2013, we reported an alternative property of zacopride which is independent of 5-HT receptors, i.e., zacopride is proved a selective *I*_K1_ /Kir2.1 channel agonist and shows antiarrhythmic effect in an aconitine-treated rat model [13, 14]. Very recently, Elnakish and coworkers demonstrated that zacopride suppresses calcium overload- induced arrhythmias in human ventricular myocardium *in vitro* [15]. In the present study, we tested the potential preventative and curative effects of zacopride on cardiac ischemic arrhythmias in rat acute MI model, and further explored the underlying mechanisms mainly focusing on the *I*_K1_ channel and transmembrane potential (TMP), using native cardiomyocytes and Chinese hamster ovary (CHO) cells transfected with Kir2.x channels.

## Materials and methods

### Animals

Male Sprague-Dawley (SD) rats (age 2 months, weight 220–250 g) were provided by the National Institute for Food and Drug Control (Shijiazhuang, China). Rats were housed under standard conditions: room temperature 20–24°C, humidity 40–60%, 12:12 h light (200 lux)-dark (LD) cycles, chaw and water *ad libitum*. The investigation conformed to the guideline for the Care and Use of Laboratory Animals (NIH, No. 85–23, revised 1996) and followed the approval of Shanxi Medical University Bioethical Committee.

#### Induction of acute ischemic arrhythmias

**Fig 1:**
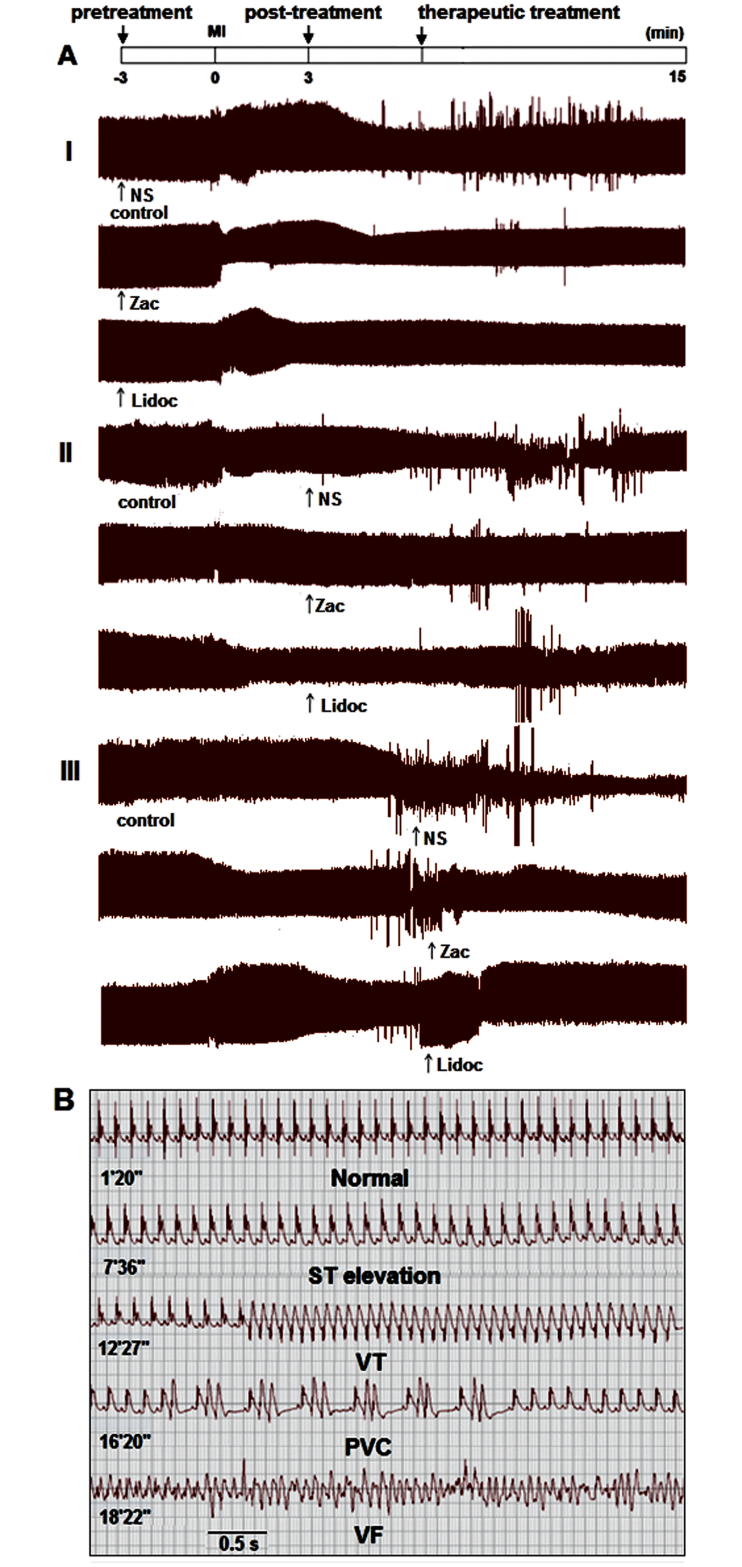
The preventative and curative effects of zacopride on MI- induced ventricular arrhythmias in anesthetized rats *in vivo*. (A) Representative condensed ECGs (25 s/div). I, pretreatment; II, post-treatment; III, therapeutic treatment. (B) Time course of ECG traces for saline control rats (coronary occlusion + saline *i*.*v*.). Zac, zacopride, at 15 μg/kg. Lidoc, lidocaine, at 7.5 mg/kg.

*In vivo study*. Arrhythmias were induced by ligating the left main coronary artery as previously described [16]. Briefly, rats were anesthetized with sodium pentobarbital (65 mg/kg, *i*.*p*.), then were intubated and ventilated with room air using an animal respirator (DH-1, Chengdu Instrument Factory, Chengdu, China) with a tidal volume of 30 ml/kg body weight and a rate of 60 tidals/min to maintain normal blood pO_2_, pCO_2_ and pH. Body temperature was maintained with an air conditioner and an appropriate heating lamp. Left thoracotomy was performed in the fourth intercostal space, and lead II electrocardiogram (ECG) was recorded throughout the experiments. After opening the pericardium, a 6–0 suture was placed around the proximal portion of the left coronary artery, and the artery wasligated for 15 minutes (min). The treatment protocols are shown in Fig 1A. In the pre-treatment group, zacopride at 5, 15, 50 μg/kg or lidocaine at 7.5 mg/kg was dissolved in 0.2 ml of saline and administered intravenously (*i*.*v*.) 3 min before coronary artery occlusion. Post-treatment with zacopride (15 μg/kg) was executed 3 min after coronary occlusion. For the therapeutic treatment of ventricular tachyarrhythmias, zacopride (15 μg/kg) was *i*.*v*. infused after the onset of the first sustained VT or VF, which usually appeared over 30 seconds (s) after coronary occlusion in our model. Each control rat received 0.2 mL saline (*i*.*v*.). Lidocaine (7.5 mg/kg) was used as a positive control drug.

*Ex vivo study*. Ventricular arrhythmias were induced by ligating the left main coronary artery in the isolated and Langendorff-perfused rat hearts as previously described [17]. In brief, rats were anesthetized with sodium pentobarbital (65 mg/kg, *i*.*p*.) and heparinized (1000 U/kg, *i*.*p*.) for 15 min. Hearts were then quickly harvested and mounted on an 80 cm H_2_O high Langendorff aortic retrograde perfusion system. Tyrode’s solution was bubbled with 100% O_2_, and the temperature was maintained at 37°C. Three ECG leads were placed on the cardiac apex, right auricule and ground to simulate ECG lead II. The ECGs were recorded with a BiopacSystem (RM6240, Chengdu Instrument Factory, Chengdu, China). All hearts were initially equilibrated for 1 hour before ECG recordings. The left main coronary artery was ligated across a small cotton roll for 15 min within 2 mm from the artery emergence and adjacent to the left atrium. Zacopride at 1 μmol/L or a combination of 1 μmol/L zacopride and 1 μmol/L BaCl_2_ was applied 3 min before coronary occlusion.

### Evaluation of arrhythmias

Prior to and during ischemia, ECGs were continuously recorded with a waveform data analysis software (RM6240, BiopacSystem, Chengdu Instrument Factory, Chengdu, China). The ventricular ectopic activity was evaluated according to the diagnostic criteria advocated by Lambeth Convention [18]. The ECGs were analyzed to determine the onset of the individual episode of arrhythmias, total episodes and episode durations of ventricular tachyarrhythmias, including premature ventricular contraction (PVC), VT and VF. Sustained VT (susVT) or VF (susVF) was designated if the lasting time of individual episode was longer than 30 s.

### Isolation of rat single ventricular myocytes

Single left ventricular myocytes were isolated using an enzymatic dissociation procedure described previously[19]. In brief, after appropriate anesthesia (sodium pentobarbital 65 mg/kg, *i*.*p*.), the rat heart was quickly harvested and placed into chilled (4°C), oxygenated (100% O_2_) and Ca^2+^-free Tyrode’s solution, then the heart was mounted onto a Langendorff retrograde perfusion apparatus via the aorta with a perfusion pressure of 80-cm H_2_O. The composition of Tyrode’s solution was (in mmol/L): NaCl 135.0, KCl 5.4, CaCl_2_ 1.8, MgCl_2_ 1.0, NaH_2_PO_4_ 0.33, HEPES 10.0, and glucose 10.0 (pH 7.3–7.4 adjusted with NaOH). The heart was first perfused with oxygenated (100% O_2_) and Ca^2+^-free Tyrode’s solution at 37°C for approximately 10 min to wash out the blood. The perfusate was then switched to enzyme-containing Tyrode’s solution and the heart was perfused for about 20 min until the tissue was adequately digested. The enzyme-containing Tyrode’s solution was composed of (in mmol/L) NaCl 125.0, KCl 5.4, MgCl_2_ 1.0, NaH_2_PO_4_ 0.33, HEPES 10.0, glucose 10.0, taurine 20.0, as well as 5.0–8.0 mg/50 mL collagenase P (Boehringe Mannheim, Germany) depending on the enzyme activity. The left ventricle was then separated and minced in Krebs buffer (KB) solution which contained (in mmol/L) KOH 85.0, L-glutamic acid 50.0, KCl 30.0, MgCl_2_ 1.0, KH_2_PO_4_ 30.0, glucose 10.0, taurine 20.0, HEPES 10.0, and EGTA 0.5. The pH was adjusted to 7.4 with KOH. The dispersed cells were filtrated twice in KB solution using a 150 μm stainless steel mesh, and then stored in KB solution at room temperature (25°C) at least 4 hours before use.

### Transfection of Kir2.x channels in Chinese hamster ovary (CHO) cells

Respective rat cardiac orthologs of Kir2.1, Kir2.2 and Kir2.3 were cloned via reverse transcriptase polymerase chain reaction (RT-PCR) and then subcloned into the eukaryotic expression vector, pEGFP-N1. CHO cells were cultured in Dulbecco’s modified Eagle’s medium supplemented with 10% fetal calf serum. Confluent CHO cells were then transiently transfected with the expression plasmids using a Lipofectamine 2000 kit (Invitrogen, USA).

### Electrophysiology

Whole-cell recording of *I*_K1_ currents in rat native ventricular myocytes and Kir2.x channel currents in CHO cells were performed under normoxic or hypoxic condition.

Briefly, isolated cardiomyocytes or Kir2.x-transfected CHO cells were transferred to a special chamber mounted on an inverted microscope (Nikon Diaphot, Nikon Co., Tokyo, Japan). The chamber was enclosed except three admittance holes intended for bath solution, 100% N_2_ and microelectrode. To create a hypoxic condition, a separate reservoir of bath solution was bubbled with 100% N_2_, and the chamber was flooded with 100% N_2_ to guarantee the pO_2_ lower than 50 mmHg (40.6 ± 0.9 mmHg) in the bath solution. The pO_2_ was measured by a blood gas analyzer (ABL80, Denmark). The cells were superfused with bath solution at 36°C for recording RMP and AP, or at room temperature (22–23°C) for recording *I*_K1_, ATP-sensitive potassium current (*I*_KATP_) and Kir2.x channel currents. The perfusion flow rate was 2 ml/min. Voltage-clamp or current-clamp recordings were performed with a whole-cell configuration using the Axopatch-200B patch clamp amplifier (Axon Instrument, USA). Patch electrodes were made from thin-walled glass capillaries (1.5 mm OD, Beijing Brain Research Institute) using a two-stage vertical microelectrode puller (PP-83, Narishge Scientific Instrument, Japan) with resistance of 2–5 MΩ. The current signal was filtered at 2 kHz. In all experiments, the membrane current density was measured, normalized to cell capacitance and expressed as pA/pF. The cell capacitance was measured using a method described by Axon Guide. The pClampex 8.2 programme (Axon Instrument, USA) was utilized to produce clamping commands and to record channel currents.

### Solutions

To measure the RMP and AP of ventricular myocytes, the bath solution contained (in mmol/L) NaCl 135.0, CaCl_2_ 1.8, MgCl_2_ 1.0, KCl 5.4, glucose 10.0, NaH_2_PO_4_ 0.33, and HEPES 10.0, and the pH was adjusted to 7.4 with NaOH. The pipette solution contained (in mmol/L) KCl 150.0, MgCl_2_ 1.0, EGTA 5.0, HEPES 5.0, ATP-K_2_ 3.0, the pH was adjusted to 7.3 with KOH. The membrane potentials were corrected for the liquid junction potential. To record the *I*_K1_ of ventricular myocytes, the bath solution contained (in mmol/L) NaCl 135.0, CaCl_2_ 1.8, MgCl_2_ 1.0, KCl 5.4, glucose 10.0, NaH_2_PO_4_ 0.33, HEPES 10.0, and CdCl_2_ 0.5, the pH was adjusted to 7.4 with NaOH. BaCl_2_ (0.2 mmol/L in the bath solution) was used to block *I*_K1_ channels. *I*_K1_ was determined as Ba^2+^-sensitive current. The pipette solution contained (in mmol/L) KCl 150.0, MgCl_2_ 1.0, EGTA 5.0, HEPES 5.0, ATP-K_2_ 3.0, and 4-aminopyridine (4-AP) 5.0 (pH 7.4 adjusted with KOH). To measure the *I*_KATP_ of ventricular myocytes, Tyrode’s solution was used as the bath solution. The pipette solution contained (in mmol/L) KCl 150.0, EGTA 10.0, HEPES 5.0, and the pH was adjusted to 7.3 with KOH. To measure the Kir2.x channel currents in CHO cells, the bath solution contained (in mmol/L): NaCl 136.0, KCl 5.0, CaCl_2_ 1.8, MgCl_2_ 1.0, glucose 10.0, HEPES 10.0 (pH 7.4). The pipette solution contained (in mmol/L) KCl 40.0, K-aspartate 80.0, KH_2_PO_4_ 10.0, phosphocreatine 3.0, EGTA 5.0, HEPES 5.0, ATP-Mg 5.0 (pH 7.2 with KOH).

### Induction of delayed afterdepolarization (DAD)

DAD is usually generated by intracellular Ca^2+^ overload in cardiomyocytes [20, 21]. We induced Ca^2+^ overload by hypoxia or isoproterenol (Iso) (1 μmol/L) (Sigma-Aldrich, USA). APs were elicited by a train of 5 depolarizing pulses with basic cycle length (BCL) of 100 ms, pulse duration of 2 ms and intensity of 0.8–1.2 nA delivered through the pipette. The incidence of DAD (the ratio of cells with DAD to total tested cells) was measured in the presence or absence of 1 μmol/L zacopride.

### Statistical analyses

Quantitative data were presented as mean ± SEM, and were analyzed with the ANOVA (analysis of variance) function of SPSS 17.0 software, followed by Least-Significant Difference (LSD) test. Numerical data, such as the episodes of a certain type of arrhythmias, were compared using the chi square (χ^2^) test between groups. Difference was considered statistically significant if the *P* value was less than 0.05.

## Results

### Zacopride suppresses acute ischemic arrhythmias, irrespective of use in a preventative or therapeutic treatment setting

*In vivo study*. The protocol for zacopride treatment is depicted in Fig 1A. In the pilot experiment, we set the coronary occlusion time to 5, 15, 30, 45 or 60 min. Severe ventricular tachyarrhythmias usually appeared 5–6 min after coronary occlusion and peaked at 9–12 min (Fig 1B), and most acute ischemic arrhythmia occurred within 15 min after MI. Therefore, we set the ischemia time to 15 min in all rats in subsequent experiments. Zacopride *i*.*v*. administration significantly reduced or even eliminated ischemic ventricular arrhythmias both in episode number and duration compared with the saline control, irrespective of administration time, before MI (pre-treatment), after MI (post-treatment), or 30 s after the appearance of the first sustained VT/VF (therapeutic treatment) (Fig 1, Tables 1 and 2). Note that 15 μg/kg zacopride exerted the optimal antiarrhythmic efficacy, which compared favorably with that of lidocaine (7.5 mg/kg), a classical antiarrhythmic drug.

**Table 1:** The suppressive effects of zacopride pretreatment or post-treatment on acute ischemic arrhythmias in anesthetized rats *in vivo* (mean ± SEM).

	n	Arrhythmia latency time (s)	Total episodes of PVC	Duration of VT (s)	Incidence of VT (%)	Duration of VF (s)	Incidence of VF (%)
**Pretreatment**							
Control	8	344.1 ± 21.1	149 ± 23	51.2 ± 16.1	100	6.9 ± 2.5	75
Zac 5 μg/kg	8	394.3 ± 18.2	157 ± 13	40.4 ± 5.2	100	0.7 ± 0.7 **	12.5 *
Zac 15 μg/kg	8	475.6 ± 35.0 **	45 ± 15**	2.1 ± 1.4**	25**	0 **	0 **
Zac 50 μg/kg	8	402.6 ± 36.7	38 ± 12**	9.2 ± 2.9**	75	0.2 ± 0.2 **	12.5 *
Lidoc 7.5 mg/kg	8	518.8 ± 27.8 **^#^	57 ± 17**^#^	1.2 ± 0.9**^#^	25**^#^	0.2 ± 0.2 **^#^	12.5 *^#^
**Post-treatment**							
Control	8	335.6 ± 11.8	105 ± 18	51.9 ± 12.3	100	4.4 ± 1.7	75
Zac 15 μg/kg	8	447.1 ± 26.1 **	14 ± 5**	1.8 ± 0.9**	37.5*	0 **	0**
Lidoc 7.5 mg/kg	8	430.6 ± 20.9**^#^	53 ± 19**^#^	1.8 ± 1.0**^#^	50^#^	0.3 ± 0.3 **^#^	12.5 *^#^

PVC, premature ventricular contraction. VT, ventricular tachycardia. VF, ventricular fibrillation. Zac, zacopride. Lidoc, lidocaine. Duration of VT, mean episode duration of VT. Duration of VF, mean episode duration of VF.

* *P* < 0.05,

** *P* < 0.01 *vs*. control.

^#^ *P* > 0.05 *vs*. 15 μg/kg Zac.

**Table 2:** The suppressive effects of zacopride therapeutic treatment on acute ischemic arrhythmias in anesthetized rats *in vivo* (mean ± SEM).

	n	Latency time of the first susVT/VF (s)	Termination time of the first susVT/VF (s)	The remaining episodes of PVC	Total duration of the remaining VT (s)	Total duration of the remaining VF (s)
Control	8	428.9±30.3^#^	129.8±54.5	110±21	191.4±55.5	30.1±11.0
Zac 15 μg/kg	8	465.1±53.3	27.5 ±5.5*	26±10**	51.9±10.9**	0**
Lidoc 7.5 mg/kg	8	494.1±44.0^#^	26.5±7.3*^#^	20±5**^#^	40.4±11.8**^#^	0.5±0.3**^#^

Latency time of the first susVT/VF, the time from coronary occlusion to the onset of the first sustained VT or VF. Sustained VT or VF was designated a sustained time > 30 s. Termination time of the first susVT/VF, the time from drug administration to the termination of the first sustained VT or VF. Total duration of the remaining VT/VF (s), total duration (s) of the remaining VT/VF during the period from the termination of the first sustained VT/VF to the 15th min after coronary occlusion.

* *P* < 0.05,

** *P* < 0.01 *vs*. control,

^#^ *P* > 0.05 *vs*.15 μg/kg Zac.

**Fig 2:**
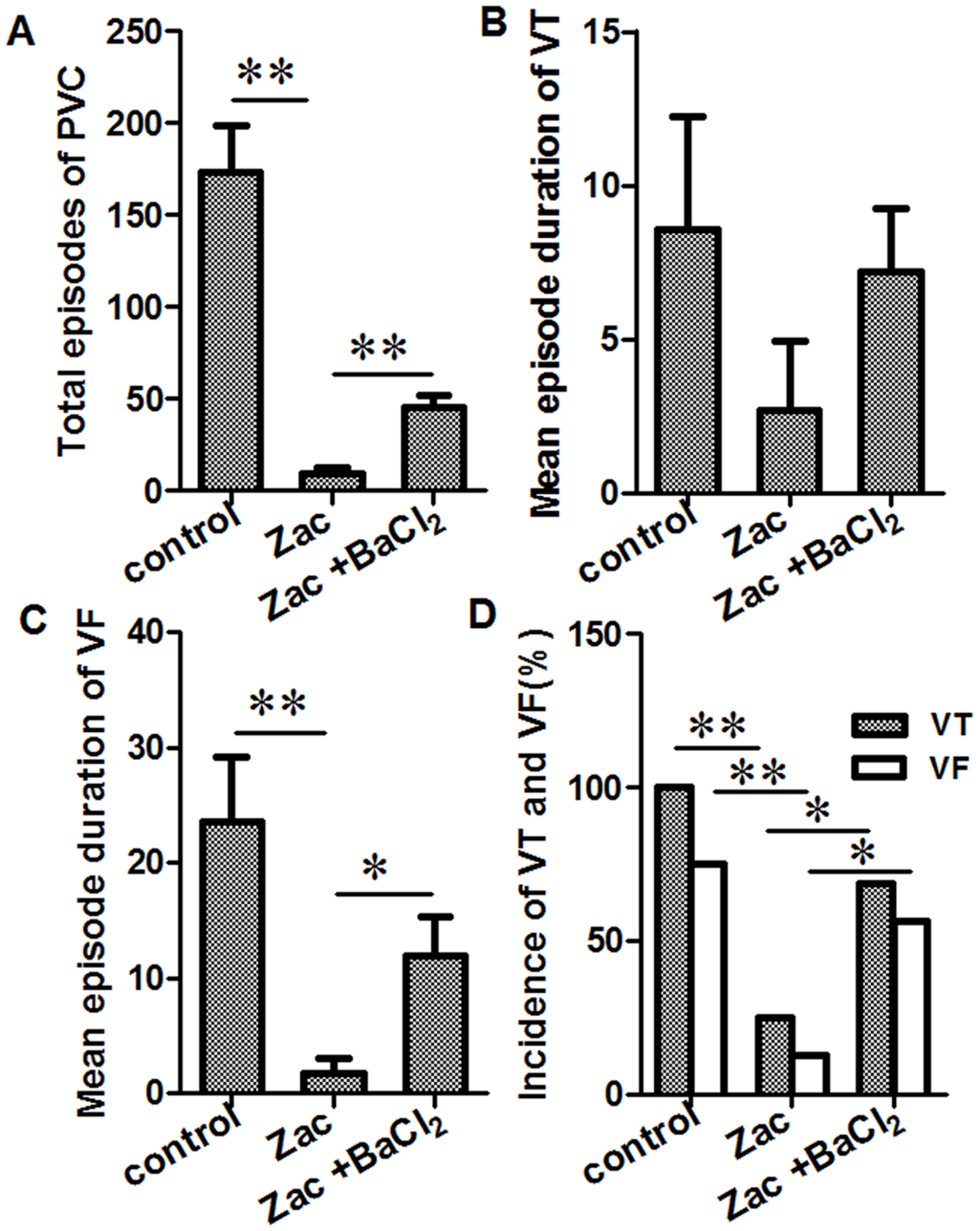
Effects of zacopride on ischemia-induced ventricular tachyarrhythmias in the *ex vivo* rat hearts. Zacopride at 1 μmol/L decreased the total number of PVC episodes (A), the mean episode duration of VT (B) or VF (C), and the incidence of VT or VF (D). BaCl_2_ at 1 μmol/L partially reversed the effect of zacopride. Zac, zacopride. * *P* < 0.05, ** *P* < 0.01.

*Ex vivo study*. According to our previously published study [13], zacopride is a selective agonist of *I*_K1_, with the optimal concentration at 1 μmol/L. Additionally, BaCl_2_, a blocker of *I*_K1_ channel, roughly abolished zacopride-mediated enhancement of the *I*_K1_ current at 1 μmol/L [13]. In the present study, data from Langendorff- perfused hearts (Fig 2A) showed that, during the 15-min ischemia in saline control rats, the mean numbers of PVC were 173 ± 26, all rats (16/16, 100%) exhibited VT, the mean episode duration of VT was 8.6 ± 3.7 s, 75% (12/16) of rats exhibited unsustained VF with a mean episode duration of 23.5 ± 5.7 s. In rats pretreated with 1 μmol/L zacopride, the number of PVC episodes were reduced to 9 ± 4, and the incidence of VT and VF decreased to 25% and 12.5%, respectively; the episode durations of VT and VF decreased respectively to 2.7 ± 2.2 s and 1.7 ± 1.3 s. BaCl_2_ at 1 μmol/L blunted the anti-arrhythmic effects of zacopride, as shown by increases in the total number of PVC episodes (45 ± 7), the mean episode duration of VT (7.2 ± 2.0 s) and VF (11.9 ± 3.5 s), and the episode frequency of VT and VF (68.8% and 56.3%, respectively) relatively to zacopride treatment. Note that zacopride or BaCl_2_ *per se* had no effects on heart rhythm in normal rats (Fig A in S1 Dataset). These results indicate that zacopride pretreatment exerts significant anti-arrhythmic effects in MI rats, and these effects are mediated by the enhancement of *I*_K1_ currents.

### Zacopride restores hypoxia-induced *I*_K1_ decline, RMP depolarization and APD prolongation in rat ventricular myocytes

**Fig 3:**
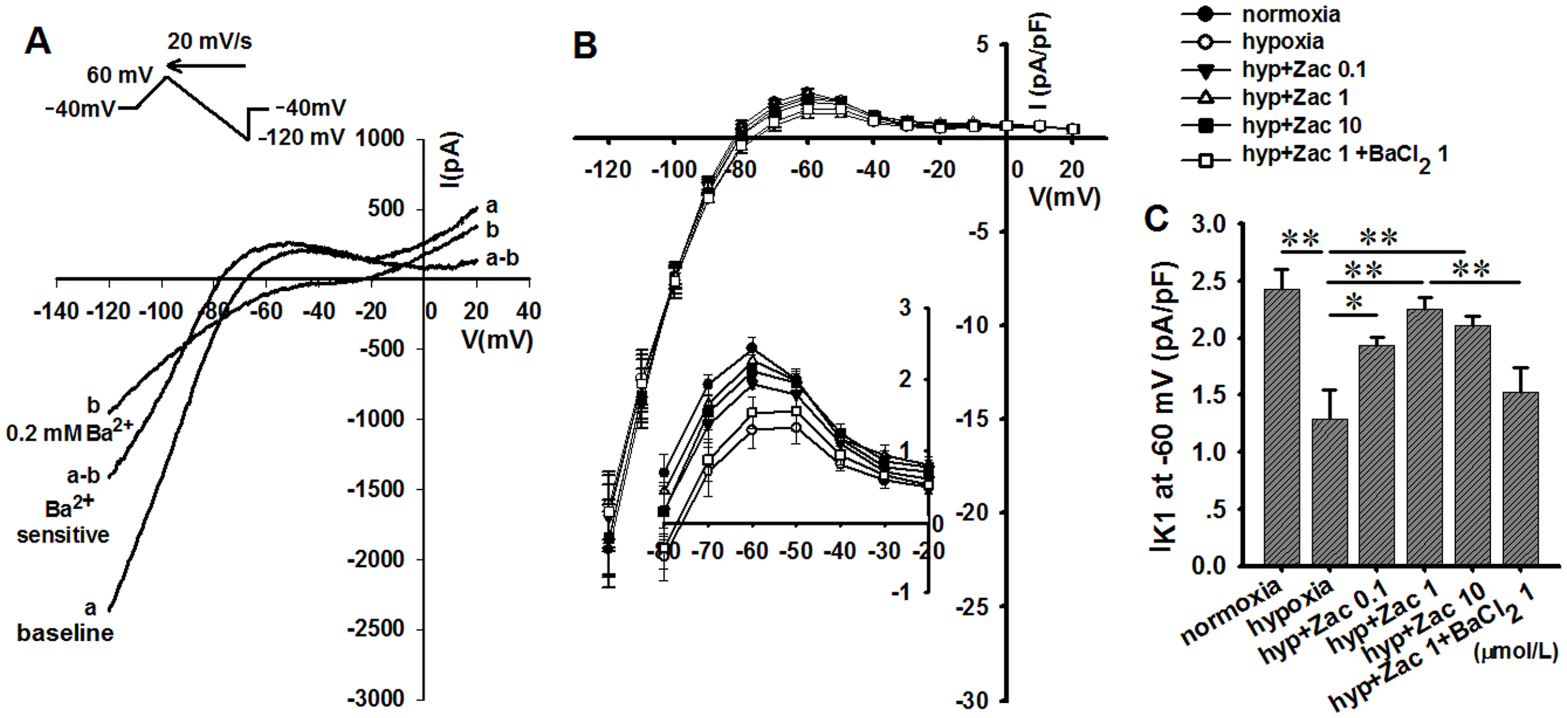
The effect of zacopride on the *I*_K1_ of hypoxic native rat ventricular myocytes. (A) A typical recording for *I*_K1_. (B) The current-voltage (I-V) curves of *I*_K1_. All traces indicated Ba^2+^-sensitive currents and were normalized for cell capacitance. The inset shows an enlarged outward portion of the I-V curves. (C) The outward *I*_K1_ currents (at –60 mV) decreased soon after the onset of hypoxia and were restored by zacopride. The maximal efficacy appeared at 1 μmol/L and attenuated by 1 μmol/L BaCl_2_. Zac, zacopride; hyp, hypoxia. * *P* < 0.05, ** *P* < 0.01.

The electrophysiological experiments which carried out under hypoxic conditions were designed to clarify the cellular mechanisms of zacopride against arrhythmias. Glibenclamide (10 μmol/L) was used in the bath solution to prevent the opening of *I*_KATP_ channel which usually occurs during hypoxia. Under the N_2_ mediated hypoxia, the *I*_K1_ current was measured with a ramp voltage-clamp pulse depolarized from a holding potential of –40 mV to +60 mV, then hyperpolarized to –120 mV at a rate of 20 mV/s. All current traces are presented as Ba^2+^-sensitive currents. As shown in Fig 3, soon after N_2_ overflowing, *I*_K1_ markedly decreased (from 2.4 ± 0.2 to 1.3 ± 0.3 pA/pF at –60 mV, n = 6, *P* < 0.01), and was restored by 1 μmol/L zacopride with a mean increase of 76.9% in the outward current (2.3 ± 0.1 pA/pF at –60 mV, n = 6, *P* < 0.01). The *I*_K1_ increase by zacopride was reversed to 1.5 ± 0.2 pA/pF at –60 mV by co-application of 1 μmol/L BaCl_2_. These results are consistent with our previous observation under normoxic condition [13].

**Fig 4:**
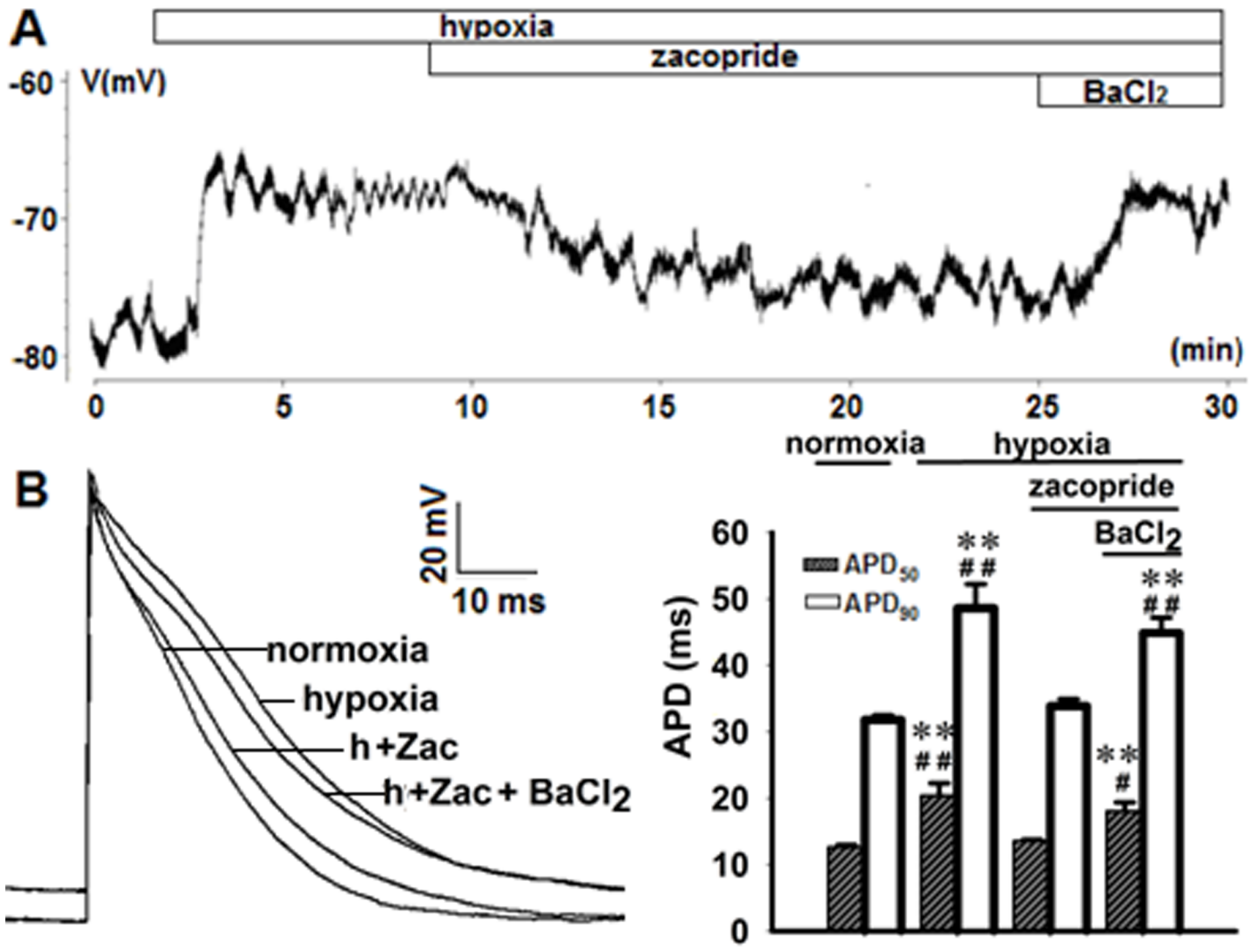
Representative transmembrane potentials (TMPs) showing the effects of zacopride on the RMP (A) and AP (B) in rat ventricular myocytes under hypoxic conditions. The right panel of B shows the statistical analysis of APD changes in response to zacopride (1 μmol/L) or zacopride (1 μmol/L) plus BaCl_2_ (1 μmol/L). Zac, zacopride. h, hypoxia. N = 6. ** *P* < 0.01, *vs*. normoxia. ^#^ *P* < 0.05, ^##^ *P* < 0.01, *vs*. Zac.

A sharp RMP depolarization occurred within the first 2 min of hypoxia, accompanying with prolongation of ADP_50_ and APD_90_ and fall of the AP amplitude (APA). Zacopride (1 μmol/L) moderately hyperpolarized the RMP and restored the ADP_50_, APD_90_ and APA. These effects were abolished by 1 μmol/L BaCl_2_ (Fig 4 and Table A in S1 Dataset).

### Zacopride does not affect hypoxia-activatated *I*_KATP_ channel

**Fig 5:**
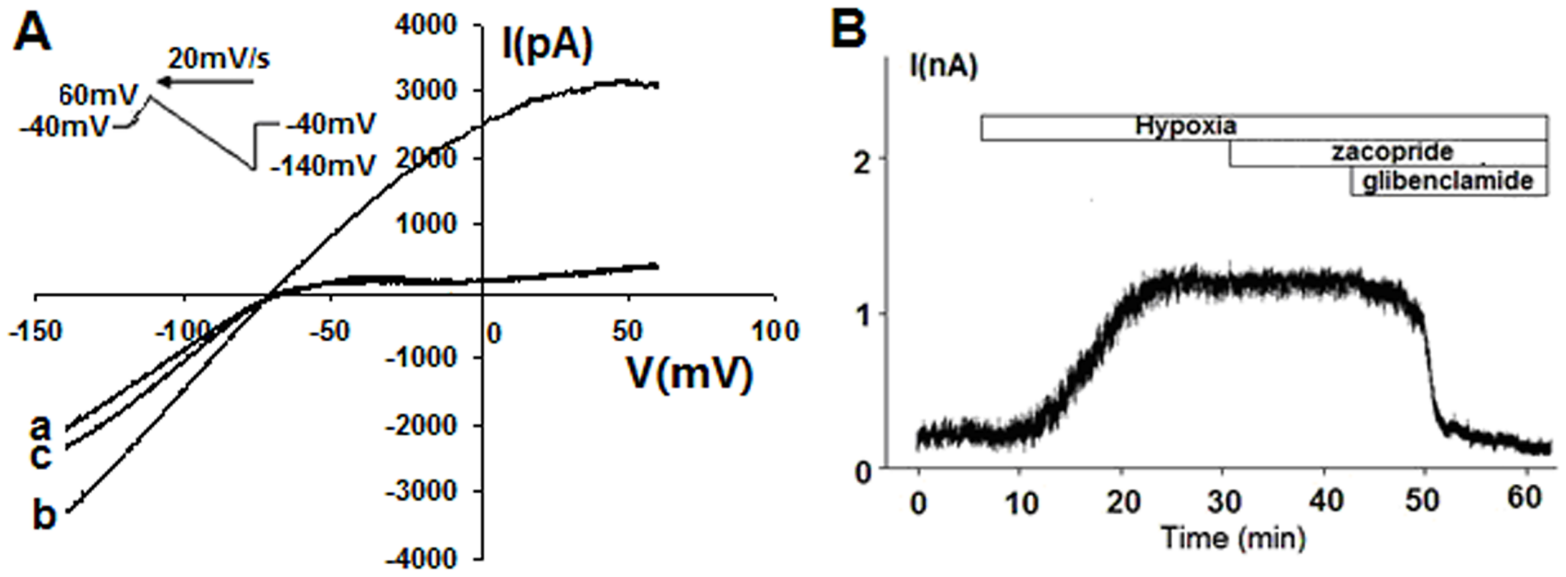
Zacopride did not affect hypoxia-induced and glibenclamide-sensitive currents in isolated rat ventricular myocytes. (A) The I-V curves of the measured currents. a, baseline. b, hypoxia. c, 10 μmol/L glibenclamide. (B) Time course of the membrane currents measured at 20 mV under hypoxic condition. One μmol/L zacopride and 10 μmol/L glibenclamide were applied in succession.

The background membrane current in response to depolarizing or hyperpolarizing voltage was recorded with a ramp voltage-clamp pulse from a holding potential of –40 mV to +60 mV, followed by hyperpolarization to –140 mV at a rate of 20 mV/s (Fig 5A). When the cell was subjected to N_2_ overflow for approximately 15 min, the outward current evidently increased at potentials positive to the RMP. Activation of the *I*_KATP_ channel was defined by a measurable increase in the outward current and inhibited by a specific *I*_KATP_ blocker glibenclamide. Under the same hypoxic condition, the cardiomyocyte was clamped at potential of +20 mV (Fig 5B). After the activation of glibenclamide-sensitive current, zacopride at 1 μmol/L did not alter the value of this current (Fig 5B).

### Zacopride suppresses hypoxia- or isoproterenol-induced DADs in rat cardiomyocytes

**Fig 6:**
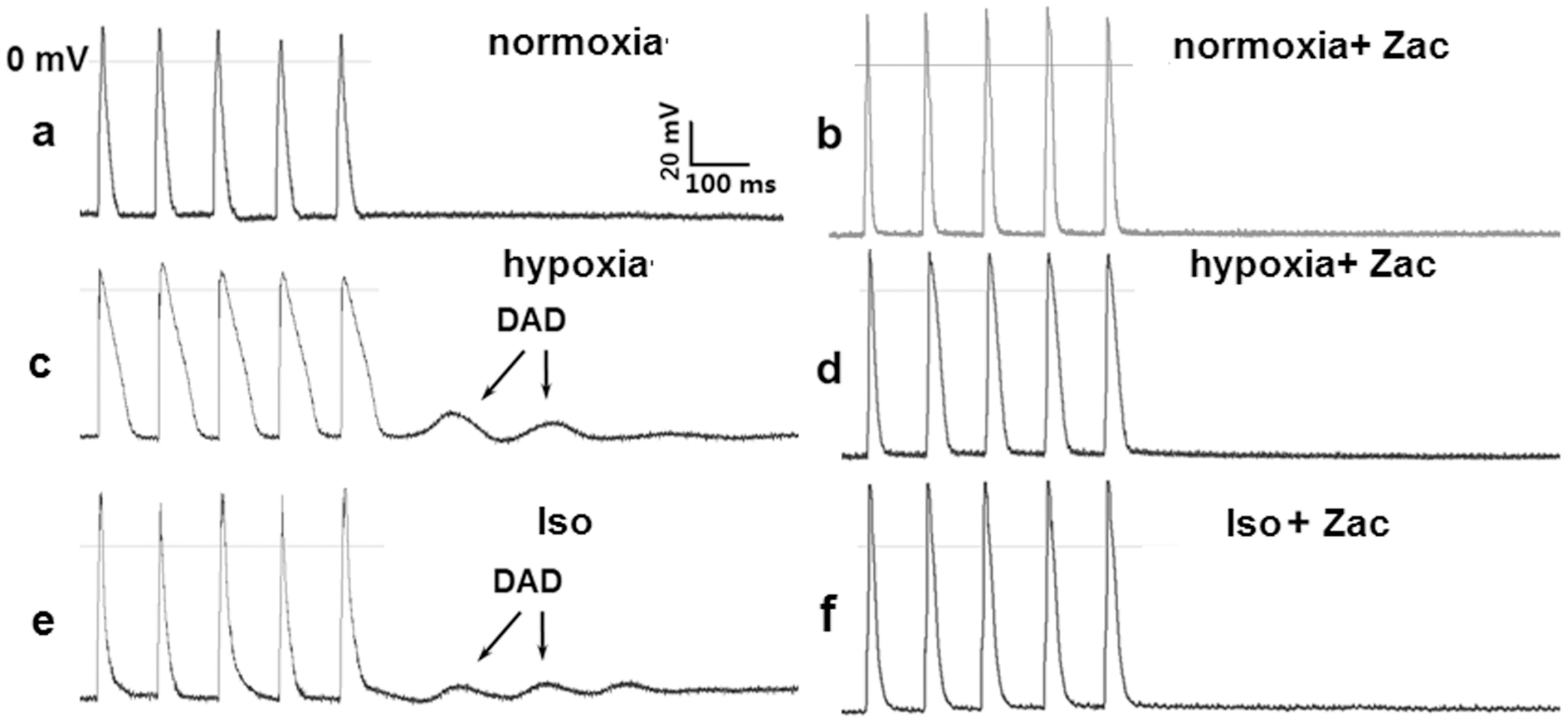
Representative transmembrane potential (TMP) traces showing hypoxia- or Iso-induced DADs in rat ventricular myocytes. a, normoxia. b, normoxia + Zac (1 μmol/L). c, hypoxia. d, hypoxia + Zac (1 μmol/L). e, Iso (1 μmol/L). f, Iso (1 μmol/L) + Zac (1 μmol/L). Zac, zacopride. Iso, isoproterenol.

At in vitro condition, cardiomyocytes lack endogenous neurohumoral regulation. To mimic ischemia and adrenergic stimulation at the cellular level in vitro, isolated cardiomyocytes were exposed to hypoxia or 1 μmol/L Iso. In the current clamp mode, an electrical train of 5 stimuli at 100 ms BCL did not introduce DAD under normoxic condition, but did under hypoxia or Iso exposure (Fig 6). Pretreatment with 1 μmol/L zacopride decreased the incidence of DAD from 73.3% (11 of 15 preparations) to 20.0% (3 of 15 preparations) (*P* < 0.01) in the hypoxic model and from 66.6% (10 of 15 preparations) to 13.3% (2 of 15 preparations) (*P* < 0.01) under Iso treatment (Fig 6).

### Zacopride rescues the hypoxia-induced decrease of *I*_Kir2.1_, but not *I*_Kir2.2_ and *I*_Kir2.3_, in CHO cells

**Fig 7:**
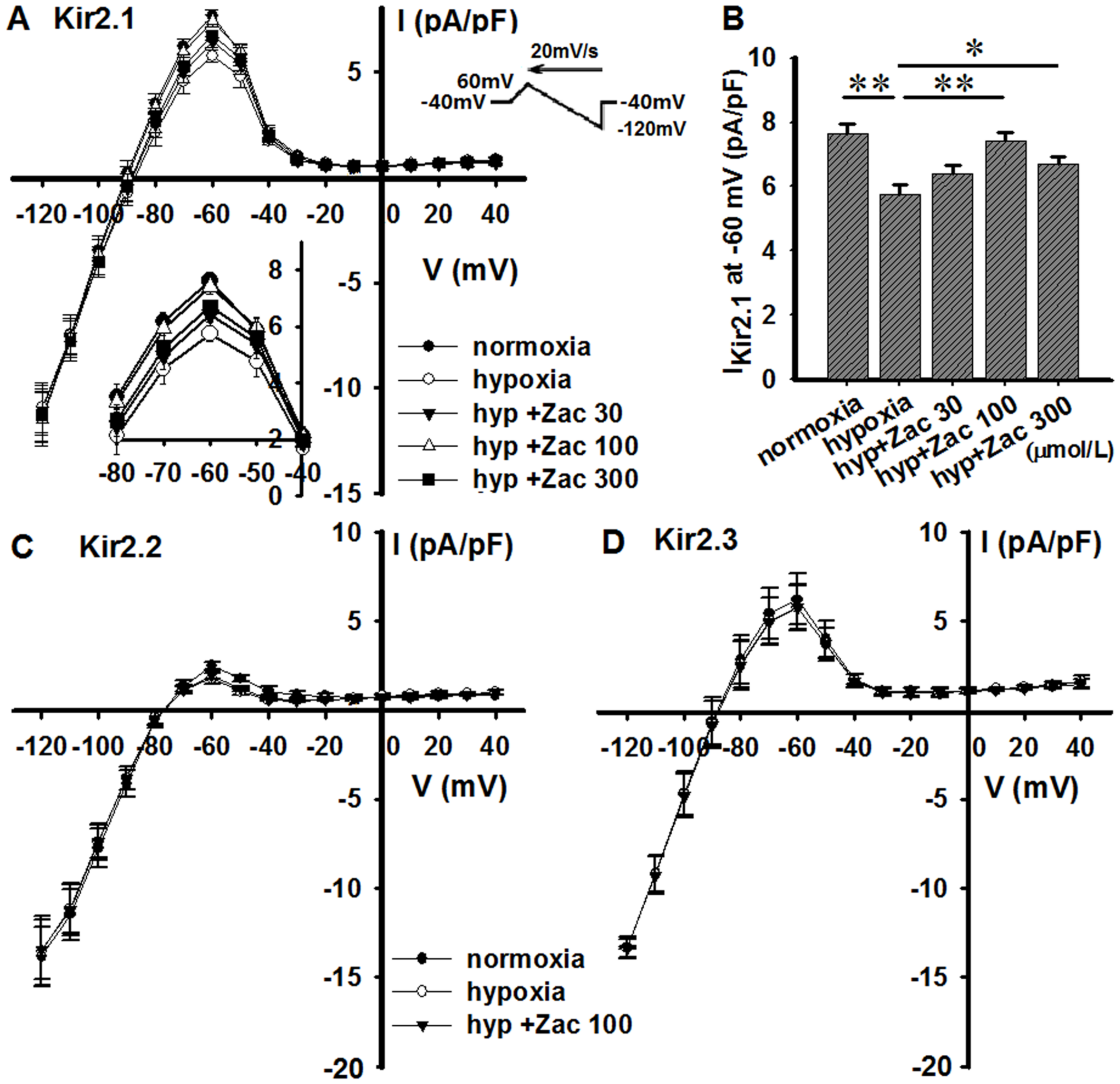
Effects of zacopride on *I*_Kir2.x_ currents in hypoxic CHO cells. **(A) and (B), Kir2.1. (C) Kir2.2. (D) Kir2.3.** The hypoxia-induced decrease of *I*_Kir2.1_ was reversed by zacopride, whereas *I*_Kir2.2_ and *I*_Kir2.3_ were refractory to both hypoxia and zacopride treatment. Zac, zacopride. Hyp, hypoxia. N = 6. * *P* < 0.05, ** *P* < 0.01.

Soon after N_2_ overflow, the outward component of *I*_Kir2.1_ decreased (from control 7.6 ± 0.3 pA/pF to 5.8 ± 0.3 pA/pF at –60 mV, *n* = 6, *P* < 0.05) in CHO cells transiently expressing Kir2.1 channels, and zacopride significantly attenuated this decrease (Fig 7A). The efficacy of zacopride was maximized at 100 μmol/L, with a mean increase of 28.8% in the outward current (from 5.8 ± 0.3 to 7.4 ± 0.3 pA/pF at –60 mV, n = 6, *P* < 0.01) (Fig 7B). Zacopride was likely less effective at 300 μmol/L than at 100 μmol/L (Fig 7B) and did not affect the inward component of *I*_Kir2.1_. Hypoxia *per se* or zacopride (100 μmol/L) did not significantly affect the *I*_Kir2.2_ or *I*_Kir2.3_ (Fig 7C and 7D). Based on these results, zacopride is likely a selective agonist of the Kir2.1 channel.

## Discussion

In the present study, we demonstrated that zacopride, a selective *I*_K1_ channel agonist, significantly eliminated acute ischemic arrhythmias in MI rats, irrespective of the time of administration (before MI, after MI, or after the occurrence of malignant arrhythmias). The underlying mechanisms are ascribed to the activation of the *I*_K1_/Kir2.1 channel, maintenance of the RMP, moderate shortening of the APD and the suppression of DADs. This study is the first one to strengthen the hypothesis that enhancing the *I*_K1_ and thereby maintaining the RMP and shortening the APD is an effective and even powerful strategy to eliminate acute ischemic arrhythmias in both preventative and therapeutic aspects. The occurrence of lethal ventricular arrhythmias in acute MI is usually unpredictable. Thus, zacopride may potentially be developed as an anti-arrhythmic drug that prevents and treats arrhythmias in acute MI.

### Zacopride restores the RMP and shortens the APD by activating the *I*_K1_/Kir2.1 channel during hypoxia or ischemia

During the acute stage of MI, K^+^ disturbance is an important event among variety of ion changes [22]. In the heart, [K^+^]_o_ is frequently elevated due to accumulation of extracellular K^+^. Increased [K^+^]_o_ and simultaneous inward currents involving Na^+^, Ca^2+^ and Cl^−^channels and exchangers depolarize the membrane [23, 24]. The conductance of the *I*_K1_ channel is increased by higher [K^+^]_o_ [25] but decreased by lysophosphatidylcholine (LPC) [6], intracellular acidosis [10, 11] and adrenergic stimulation[12]. Eventually, the *I*_K1_ conductance may decrease and lead to membrane potential depolarization. During cardiac ischemia, changes in APD involve an initial lengthening followed by a marked shortening[26, 27]. The APD prolongation upon early ischemia is due to a reduction of electrogenic pump current, a fall of *I*_K1_ as a consequence of intracellular acidosis, and an acute inhibition of *I*_to_[27]. The shortening of APD is partially attributable to activation of *I*_KATP_ [27, 28].

In the present study, we created a hypoxic environment by N_2_ flux around patched cells in an enclosed chamber, and this manipulation reduced the pO_2_ of the bath solution to less than 50 mmHg. The RMP depolarization was well recognized within the first minute of hypoxia, along with *I*_K1_ reduction and APD prolongation during the early stage of hypoxia. Zacopride treatment enhanced the *I*_K1,_ restored the depolarized RMP, and moderately shortened the APD. According to our previous work [13] and the present study, BaCl_2_ at 1 μmol/L could abolish zacopride (1 μmol/L)-induced *I*_K1_ increase under nomoxic or hypoxic conditions. It makes sense of subsequent results that 1 μmol/L BaCl_2_ diminished the effect of zacopride on RMP and APD during hypoxia. Because these experiments were all performed in the presence of glibenclamide (*I*_KATP_ channel blocker), we excluded *I*_KATP_ as a confounding factor when explaining the relationship between *I*_K1_ and RMP/APD.

Activation of the *I*_KATP_ channel also hyperpolarizes the RMP and shortens the APD during hypoxia or ischemia, but it may be a relatively late event[29, 30]. In the present study, the *I*_KATP_ channel opened at least 15 min after the onset of hypoxia (shown in Fig 5), an phenomenon consistent with the observation by Liu *et al* [30]. The subsequent application of 1.0 μmol/L zacopride did not affect the *I*_KATP_ channel. Combining the observation that BaCl_2_ inhibited the effects of zacopride on the RMP and APD, we conclude that the effect of zacopride on RMP and APD is mediated by *I*_K1_, rather than by *I*_KATP_.

### Enhancing RMP by activating the *I*_K1_ channel may represent a strategy to manipulate lethal arrhythmias in acute MI

Following coronary occlusion, ventricular arrhythmias are the critical cause of mortality in rats [31]. Re-entry, enhanced automaticity and triggered activity are likely the ultimate electrophysiological manifestation and the main underlying arrhythmogenic mechanisms in acute MI based on membrane damage and ionic imbalance [32]. We previously reported the ability of zacopride in attenuating aconitine-induced triggered arrhythmia [13]. In cardiac ischemia, myocardial substrates likely favor re-entry, but non-reentrant mechanisms, such as abnomal automaticity and triggered activity (TA) may be the most important initiators for lethal arrhythmias [33 – 35].

During cardiac ischemia, a burst of arrhythmias, including PVC, VT and VF, begins approximately 5 min after coronary occlusion and persists for at least 15 min in the rat model. These early arrhythmias are mainly attributable to disturbances in the RMP [4 – 6] and adrenergic activation (*in vivo*) [7 – 9]. For instance, there are an 100- fold increase in the catecholamine concentration within the extracellular space of the ischemic zone, a two fold increase in functionally coupled α-adrenoceptors and a 30% increase in β-adrenoceptors 15 min after myocardial ischemia [8]. Our hypothesis that enhancing the *I*_K1_ current attenuates arrhythmia in acute MI is based on the following three theoretical perspectives.

First, moderate enhancement of *I*_K1_ is a compensation for the reduction in this current and helps to maintain a normal RMP, the latter would likely eliminate any abnormal automaticity and improve the conductivity. During hypoxia or ischemia, cardiomyocytes are usually depolarized due to extracellular K^+^ accumulation [4] and intracellular acidosis [5]. Depolarization makes the membrane potential more prone to reach the threshold for Na^+^ channel activation and eventually generate propagated action potentials [35]. By enhancing the *I*_K1_, zacopride prevents RMP depolarization and consequently decreases cardiac excitability and autorhythmicity. Moreover, depolarization also inactivates voltage-gated Na^+^ channel. The recovery of RMP may increase Na^+^ channel availability and improve the conductivity in ischemic myocardium, and thus help to prevent or terminate re-entrant activity [36].

Second, *I*_K1_ channels determine the diastolic membrane conductance of cardiomocytes. Moderate *I*_K1_ enhancement increases the diastolic conductance and attenuates the fluctuation of membrane potential and thus to avoid aberrant action potentials aroused by a smaller depolarization [37].

Third, regarding the importance of the *I*_K1_ in maintaining the diastolic RMP and late phase 3 repolarization, enhancing *I*_K1_ is beneficial to abolishing or attenuating DADs or early afterdepolarizations (EADs). *In vivo*, Ca^2+^ overload due to ischemic membrane damage and adrenergic stimulation may initiate DADs [20]. As shown in the present study, zacopride increased the resting membrane conductance, which is a critical electrophysiological mechanism for diminishing the onset of DADs. By increasing the repolarizing current, zacopride moderately shortened the APD, which also helps to reduce Ca^2+^ influx and the development of DADs. In addition, moderate APD shortening alleviates AP triangulation and shrinks the vulnerable window for the reactivation of voltage-gated Ca^2+^ channel. Overall, both effects may alleviate the susceptibility of ventricular myocardium to EADs [38].

The *I*_KATP_ channel operates only when the intracellular ATP concentration is significantly decreased (i.e., during hypoxia or ischemia). The openers/agonists of *I*_KATP_ channel also hyperpolarize the RMP and shorten the APD, which are acknowledged to protect ischemic hearts. The emergence of the *I*_KATP_ channel agonists constitutes an advance in anti-arrhythmia efforts albeit far from totally effective. The critical reason might be that the inward rectification of *I*_KATP_ is markedly weaker than that of *I*_K1_. When the membrane potential is depolarized, activated *I*_KATP_ permits generous potassium efflux during the plateau phase of AP. The resultant visible shortening of the APD associated with the shortening of the refractory period may even promote arrhythmia. Alternatively, the *I*_K1_ channel only slightly conducts current during the plateau phase due to the strong inward rectification, which helps to maintain a long-lasting plateau phase and prevents excessive potassium loss. Therefore, Lopatin *et al* [2] predicted that the ideal drug for suppressing ventricular arrhythmias ought to have the property of opening K^+^ channels at the resting potential, but not at the peak of the action potential, i.e., one that acts on the *I*_K1_. As a specific agonist of *I*_K1_, zacopride is the very candidate. Moreover, considering the time course of membrane damage and membrane potential disturbance during ischemia, zacopride may provide protection to cardiomyocytes during the very early stage of ischemia.

### Special concerns about enhancing *I*_K1_

Upregulation of *I*_K1_ has been deemed to a risk factor for atrial arrhythmogenesis [39 – 41]. Increase of *I*_K1_ yields APD and ERP shortening, thus theoretically increases the curvature of spiral waves and thereby stabilizes rotors and re-entry arrhythmias [42, 43]. A concern remains that zacopride may increase the susceptibility to atrial fibrillation (AF) when used in the clinic for treating ischemic ventricular arrhythmias. But is that true? The native *I*_K1_ channels in the heart are assembled by Kir2.1 (KCNJ2), Kir2.2 (KCNJ12) and Kir2.3 (KCNJ4) channels, and these channels show tissue- and species-specific profiles [44, 45]. Based on the Western blot results from our previous work [14], Kir2.1 is the predominant isoform in rat ventricles, whereas Kir2.3 is the major isoform in the atrium. Kir2.1 expression in the atrium is only 25% of that in the ventricle. Our previous study in Kir2.x-transfected HEK-293 cells under normoxia [14] and the present results from the Kir2.x-cloned CHO cells under hypoxia indicate that the target channel of zacopride is *I*_Kir2.1_, whereas *I*_Kir2.2_ and *I*_Kir2.3_ channels were refractory to zacopride (shown in Fig 7). These observations explain why zacopride did not affect the atrial *I*_K1_ or APD [14] and excluded the possibility of AF occurrence from a theoretical perspective. However, because the association between zacopride and AF has not been studied, the use of zacopride in cases of AF should be approached with caution.

A second concern is the risk of ventricular re-entry arrhythmias. A significant increase in *I*_K1_ may shorten APD and ERP, and thereby promote re-entry activities. For example, in two transgenic mice models [46, 47], the *I*_K1_ conductance was strikingly increased by approximately 12–14 fold, with resultant severe ventricular arrhythmias. It seems contradictory to advocate the development of anti-arrhythmic agents that enhance *I*_K1_. However, this discrepancy may stem from the extent of *I*_K1_ regulation, which should be limited. Conditions in transgenetic model usually significantly deviate from physiology. As reported by Li *et a l* [47], the ADP_90_ of cardiac myocytes in transgenetic mice was shortened from 21.8 ms (control) to 7.9 ms and accompanied by a more than 10 fold higher density of *I*_K1_. Excessive increases in *I*_K1_ do not further hyperpolarize the RMP due to the inward rectifying property of *I*_K1_, but may markedly accelerate repolarization and shorten the APD, which is undoubtedly pro-arrhythmic. In contrast, zacopride only evokes a 33% increase in *I*_K1_ at –60 mV under normoxic condition [13], and a 76.9% increase upon hypoxia. Such a moderate *I*_K1_ increment recovered the RMP from –64.4 ± 0.7 mV to –77.4 ± 1.2 mV, which is close to physiological levels (–79.1 ± 1.3 mV). Correspondingly, the shortening of APD_50_ and APD_90_ by zacopride were less than 40%. Modulating *I*_K1_ within the physiological range is important to minimize the pro-arrhythmic effects of anti-arrhythmic medications.

### Limitations

A major limitation of the present study is species differences. The *I*_to_ current underlies the initial and rapid (phase 1) repolarization of the AP, and the *I*_K_ is mainly responsible for subsequent phase 2 and phase 3 repolarization [48]. In adult rat ventricular myocytes, the *I*_K_ current is smaller and its role is less pivotal, so larger *I*_to_ leads to a triangle-like AP morphology [49–51]. But *I*_K_ is a major determinant of plateau phase duration and the rates of phases 2 and 3 AP repolarization in human and most nonrodent mammalian cardiomyocytes [50]. *I*_K1_ is primarily involved in late phase 3 repolarization towards the RMP. Heteromultimer formation among diverse Kir2 family subunits determines the properties of *I*_K1_ in different species and consequently influences *I*_K1_ contribution to AP configuration [52, 53]. Although the results in rats support our hypothesis, further studies in other species are warranted. Recently, zacopride was reported to exert anti-arrhythmic effects in human ventricular myocardium [15]. *I*_K1_ channel may be a promising target for treating human acute ischemic arrhythmias in the future if indication, dosage and administration time are appropriately designed.

## Supporting information

S1 Dataset Supplementary data for ECG and action potential recordings.tFig A. The representative ECG traces before and after administration of zacopride (Zac) or BaCl_2_ respectively in normal isolated rat hearts. Table A. Effects of zacopride on the action potential parameters of rat ventricular myocytes.
(DOC)

S2 Dataset The original recording traces from electrophysiological experiments. (DOCX)

S3 Dataset The data tables containing all raw data for statistical analysis. (XLSX)
