# Supplementary material for: The IK1/Kir2.1 channel agonist zacopride prevents and cures acute ischemic arrhythmias in the rat
Source: PLoS One. 2017 May 18;12(5):e0177600. doi: 10.1371/journal.pone.0177600 (PMC5436763; doi:10.1371/journal.pone.0177600)
Supplement: S1 Dataset — The representative ECG traces before and after administration of zacopride (Zac) or BaCl2 respectively in normal isolated rat hearts. Table A. Effects of zacopride on the action potential parameters of rat ventricular myocytes. (DOC) [file pone.0177600.s001.doc]

**Supplementary information**

**The *I*_K1_/Kir2.1 channel agonist zacopride prevents and cures acute ischemic arrhythmias in the rat**

Xu-Wen Zhai^1^, Li Zhang^2^, Yun-Fei Guo^3^, Ying Yang^3^, Dong-Ming Wang^4^, Yan Zhang^3^, Pan Li^3^, Yi-Fan Niu^3^, Qi-Long Feng^1^, Bo-Wei Wu^1^, Ji-Min Cao^5*^, Qing-Hua Liu^3*^

^1^ Department of Physiology, Shanxi Medical University, Taiyuan, China;

^2^ Shanxi Provincial Children’s Hospital, Taiyuan, China;

^3^ Department of Pathophysiology, Shanxi Medical University, Taiyuan, China;

^4^ The Second Hospital, Shanxi Medical University, Taiyuan, China;

^5^ Department of Physiology, Institute of Basic Medical Sciences, Chinese Academy of Medical Sciences, School of Basic Medicine, Peking Union Medical College, Beijing, China

* Corresponding authors:

Email: [liuqh20041206@163.com](mailto:liuqh20041206@163.com) (QL)

Email: [caojimin@126.com](mailto:caojimin@126.com) (JC)

This work was supported by grants from the National Natural Science Foundation of China (No. 31200864 to Liu QH and No. 81670313 to Cao JM) and a grant from Shanxi Scholarship Council of China (No. 2016-059 to Liu QH)

**Competing interests**

None.

**
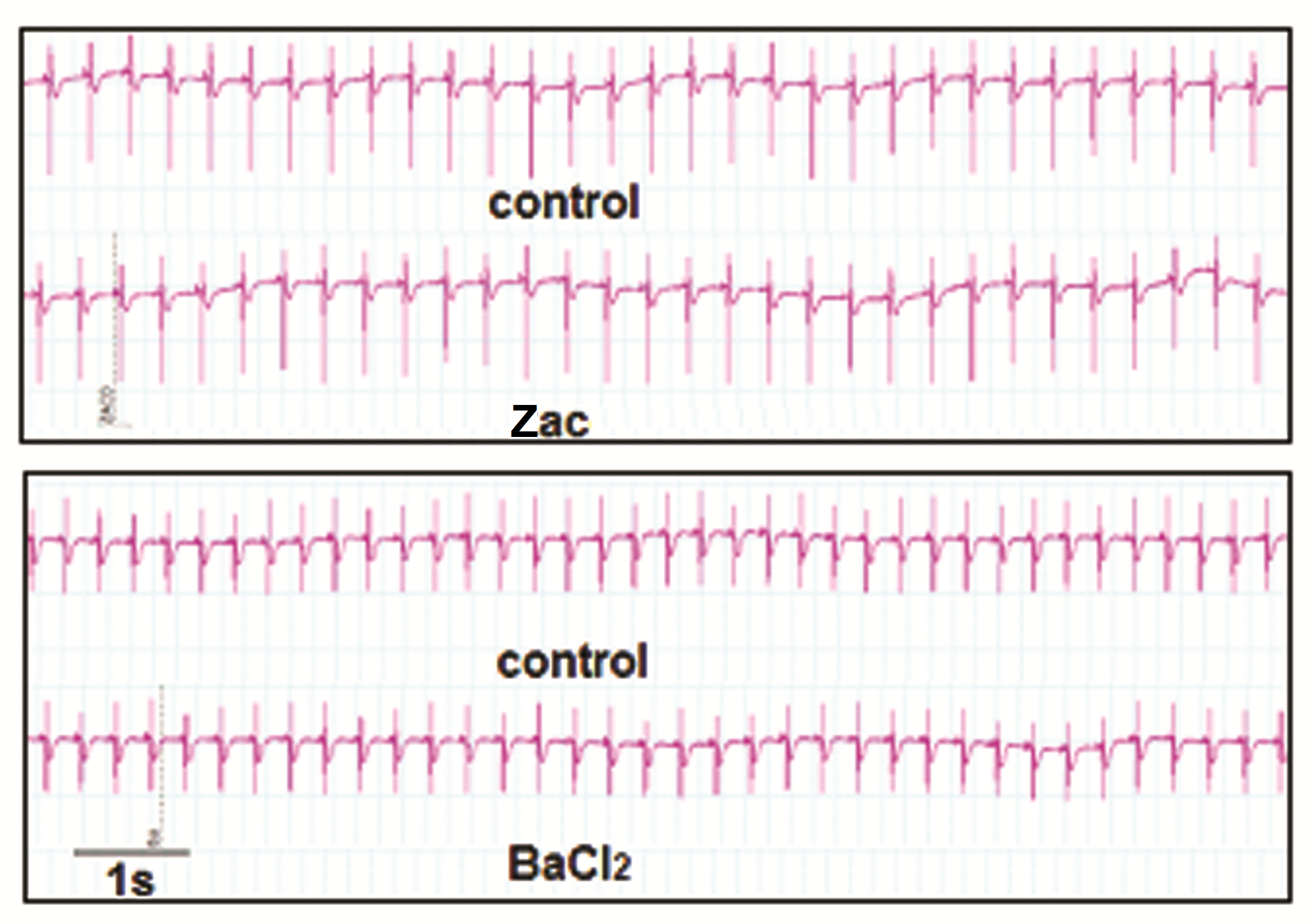
**

**Fig A.** The representative ECG traces before and after administration of zacopride (Zac) or BaCl_2_ respectively in normal isolated rat hearts. Zacopride (upper panel) or BaCl_2_ (lower panel) per se had no effect on cardiac rhythm.

**Table A. Effects of zacopride on the action potential parameters (mean ± SEM)**

|  | normoxia | hypoxia | | |
| --- | --- | --- | --- | --- |
|  |  | control | zacopride | zacopride + BaCl_2_ |
| RMP (mV) | −79.1 ± 1.3 | −64.4 ± 0.7^** ##^ | −77.4 ± 1.2 | −66.3 ± 1.0^** ##^ |
| APA (mV) | 113.8 ± 2.3 | 89.3 ± 7.4^** #^ | 109.2 ± 4.1 | 93.1 ± 7.7^*^ |
| APD_50_ (ms) | 12.6 ± 0.3 | 20.4 ± 1.9^** ##^ | 13.6 ± 0.2 | 18.1 ± 1.3^** #^ |
| APD_90_ (ms) | 31.8 ± 0.7 | 48.6 ± 3.6^** ##^ | 33.8 ± 1.1 | 44.8 ± 2.3^** ##^ |

APA, action potential amplitude. APD_50_ and APD_90_, action potential duration at 50% and 90% repolarization, respectively. RMP, resting membrane potential. The concentrations of zacopride (Zac) and BaCl_2_ were both 1 μmol/L. N = 6 in each group. ^*^ *P* < 0.05, ^**^ *P* < 0.01, *vs.* normoxia. ^#^ *P* < 0.05, ^##^ *P* < 0.01, *vs.* Zac.
