# Supplementary material for: The IK1/Kir2.1 channel agonist zacopride prevents and cures acute ischemic arrhythmias in the rat
Source: PLoS One. 2017 May 18;12(5):e0177600. doi: 10.1371/journal.pone.0177600 (PMC5436763; doi:10.1371/journal.pone.0177600)

1. **In vivo MI**

The Fig 1.E showed representative condensed electrocardiograms the ECG tracing I-III were picked up, expended and shown at the bottom of panel.

**Control**

**Pre-ligation-normal ECG**


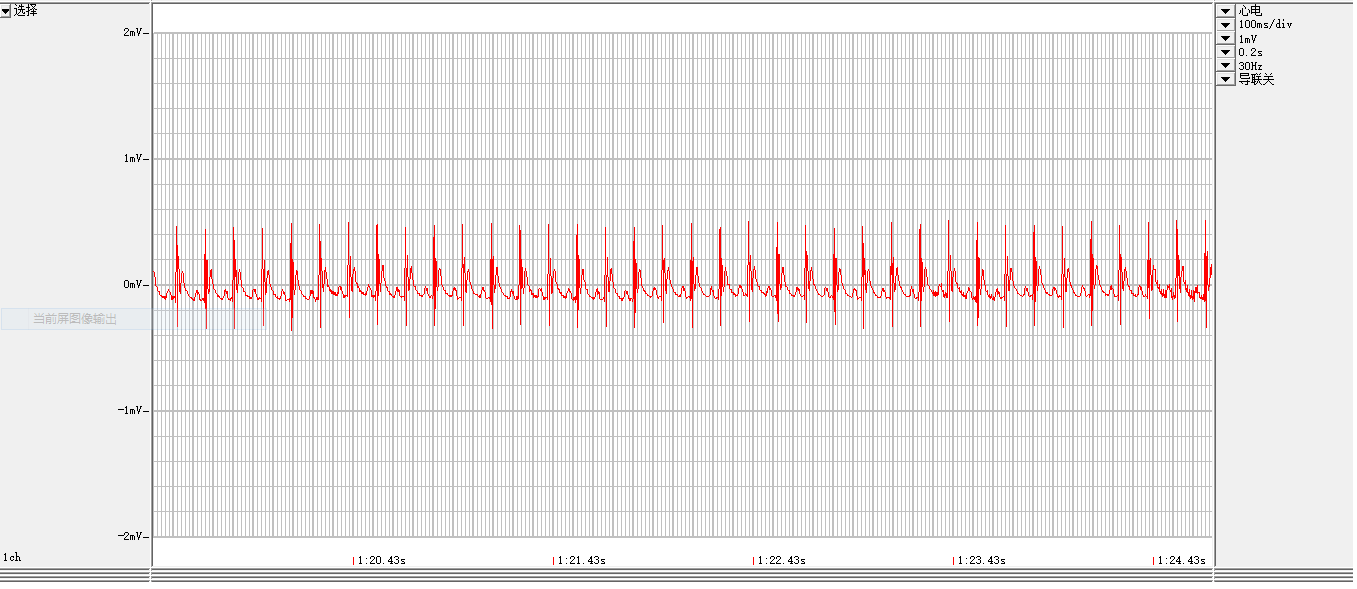


**Ligation-ST elevation**


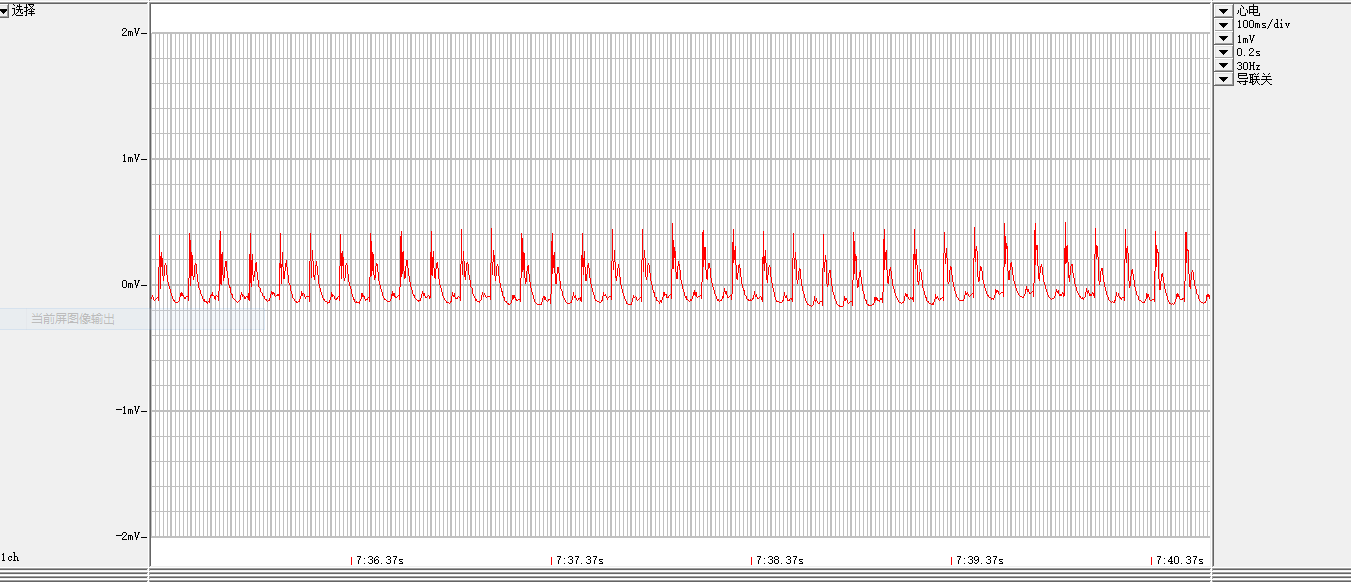


**Onset of arrhythmia, about 5 min post ligation**


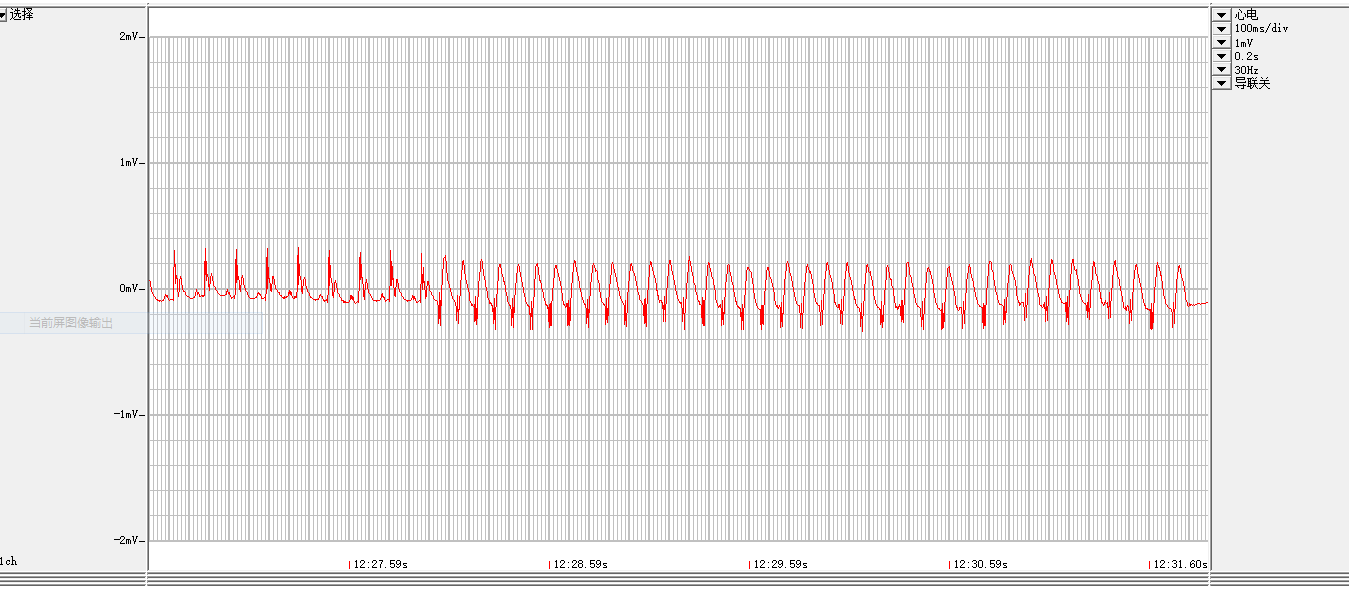


**PVC**


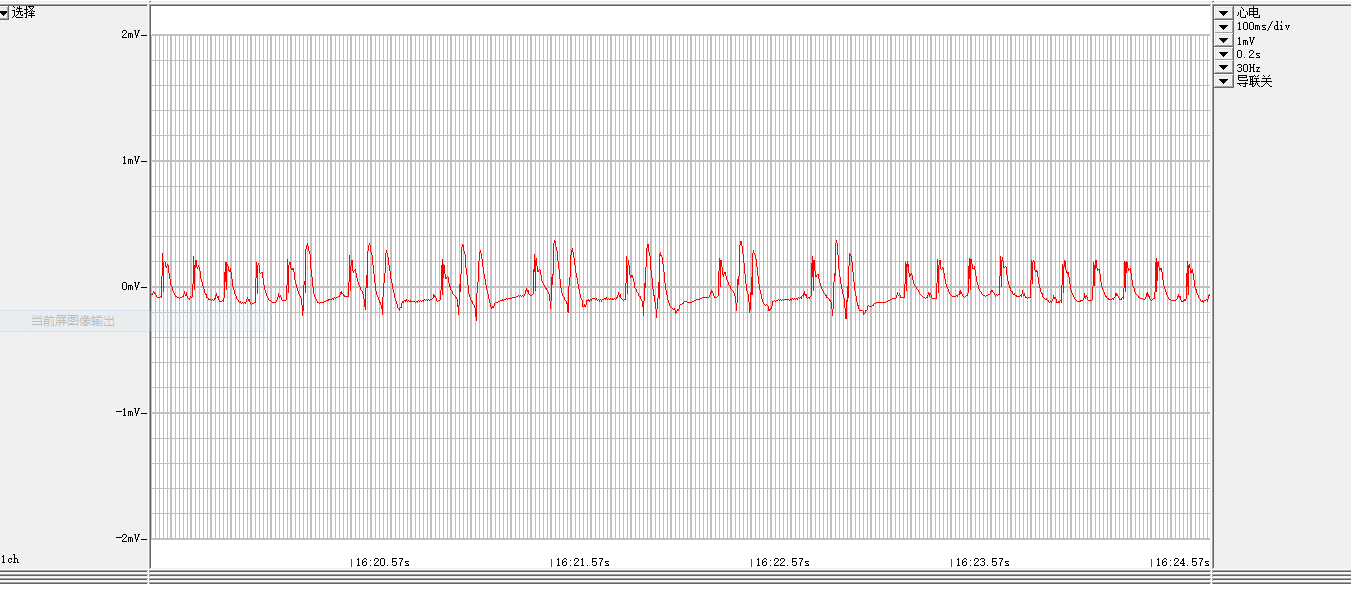


**VF**


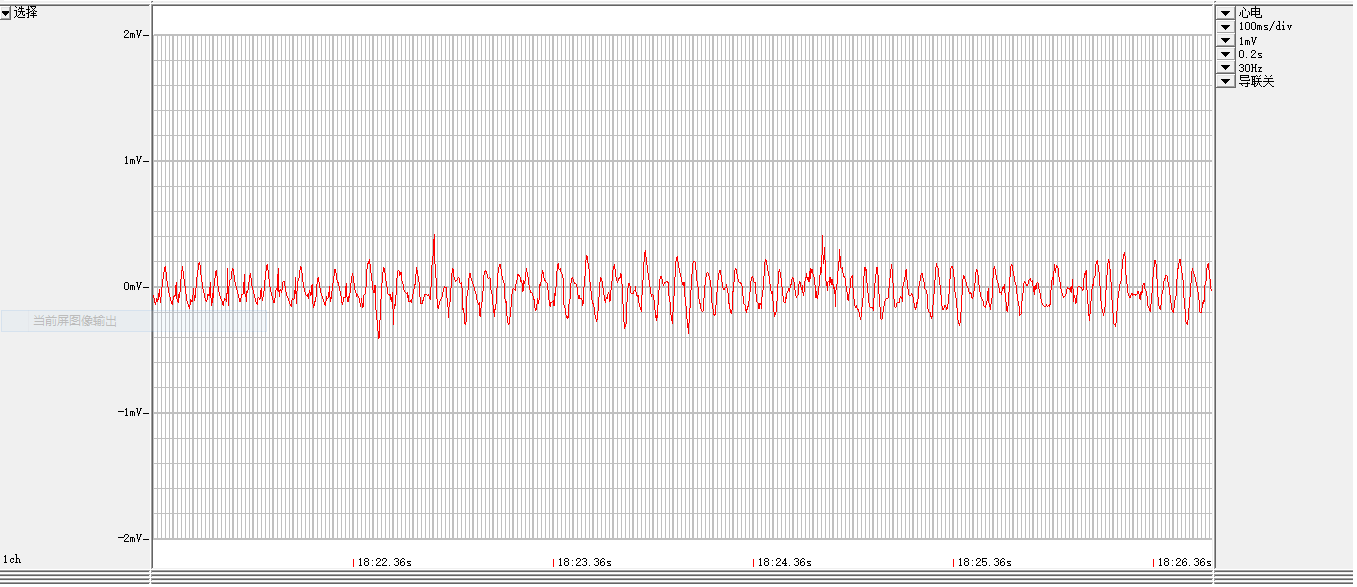


**Zacopride**

**Pre-ligation-normal ECG**

**
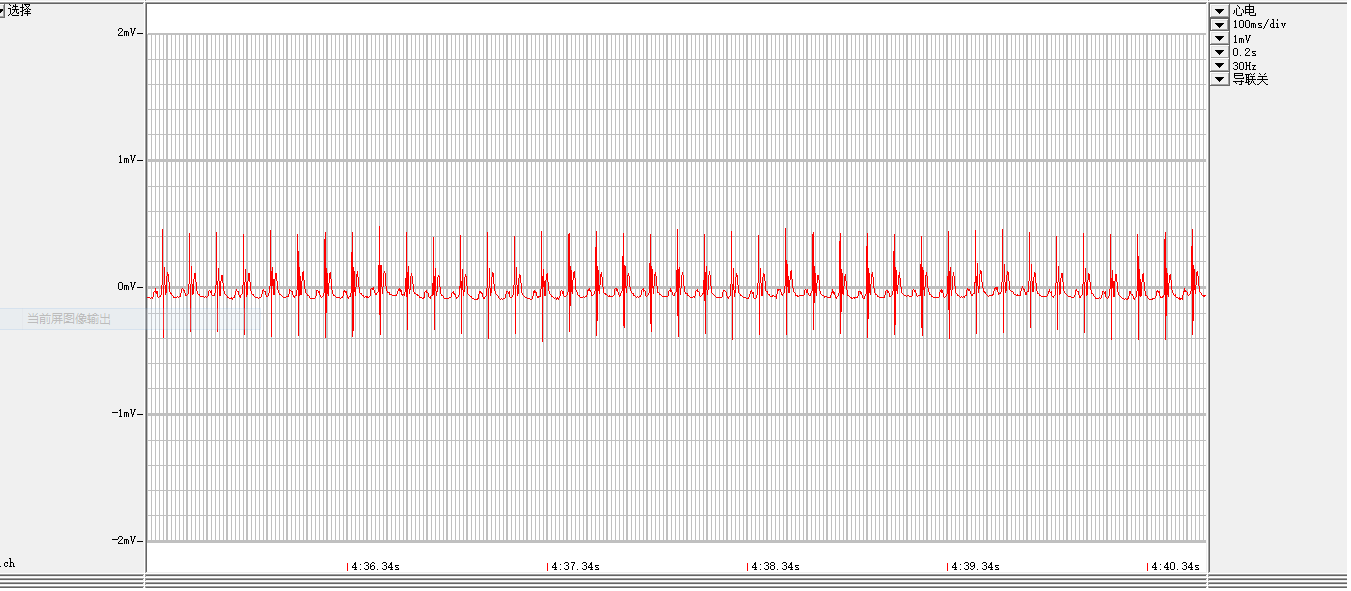
**

**Pre-ligation-Zacopride**

**
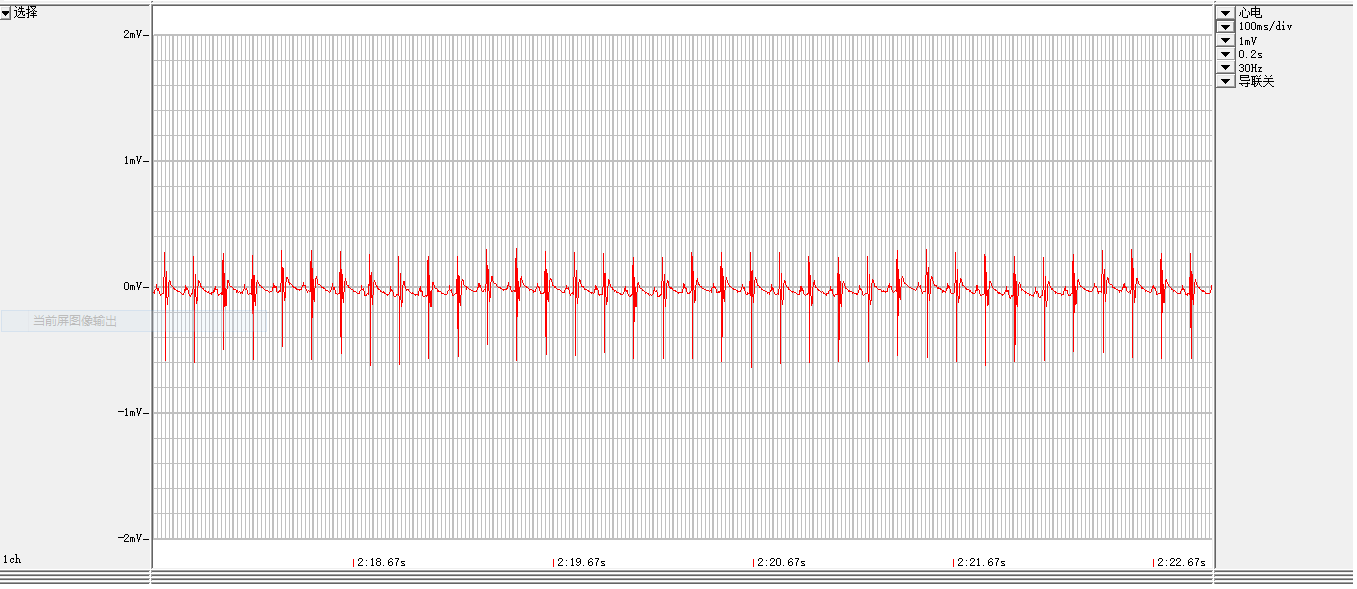
**

**Ligation-ST elevation**

**
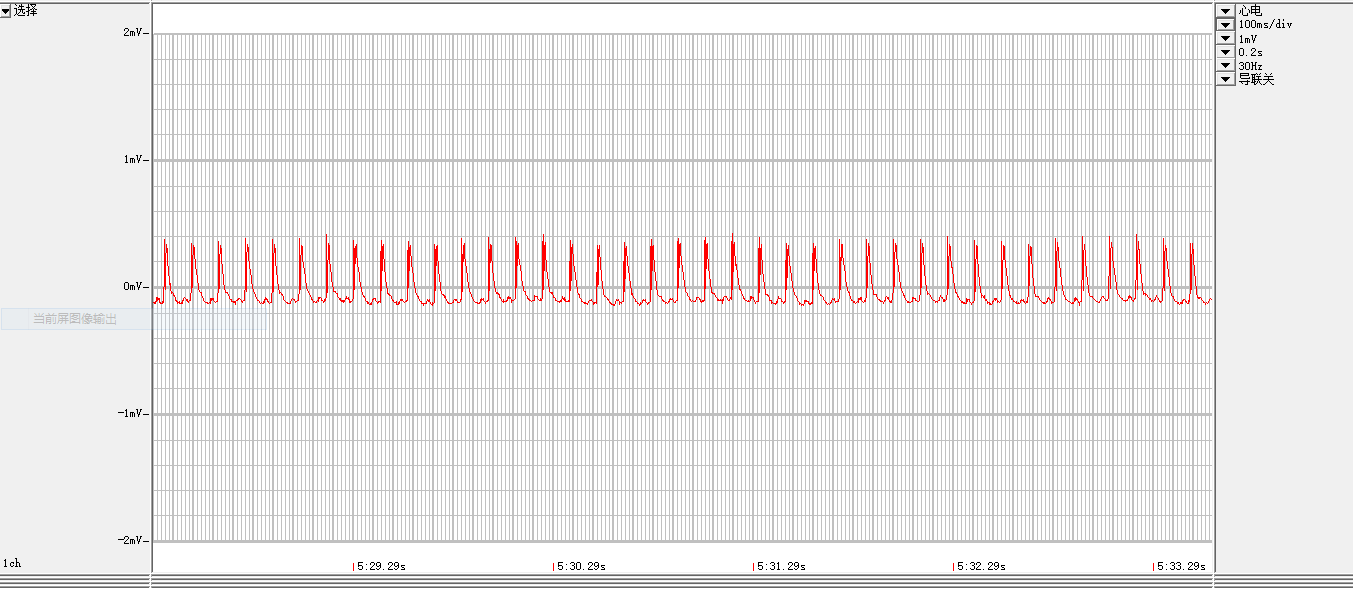
**

**Onset of arrhythmia, about 9 min post ligation**

**
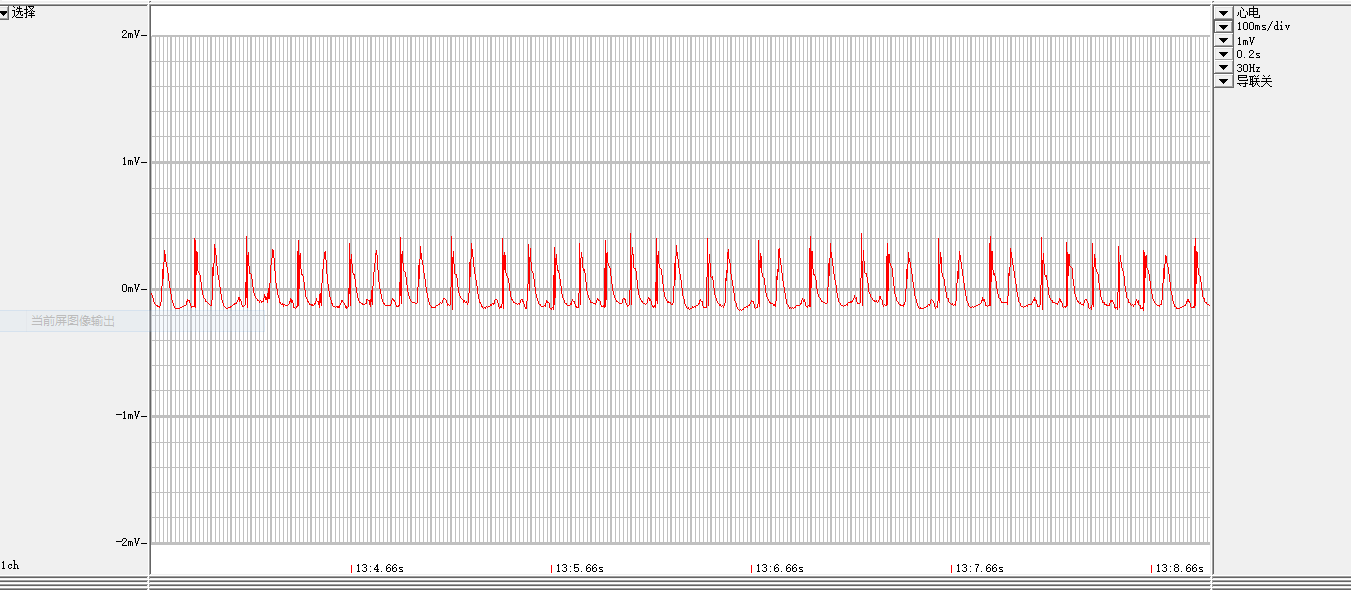
**

**Ligation-about 15min**

**
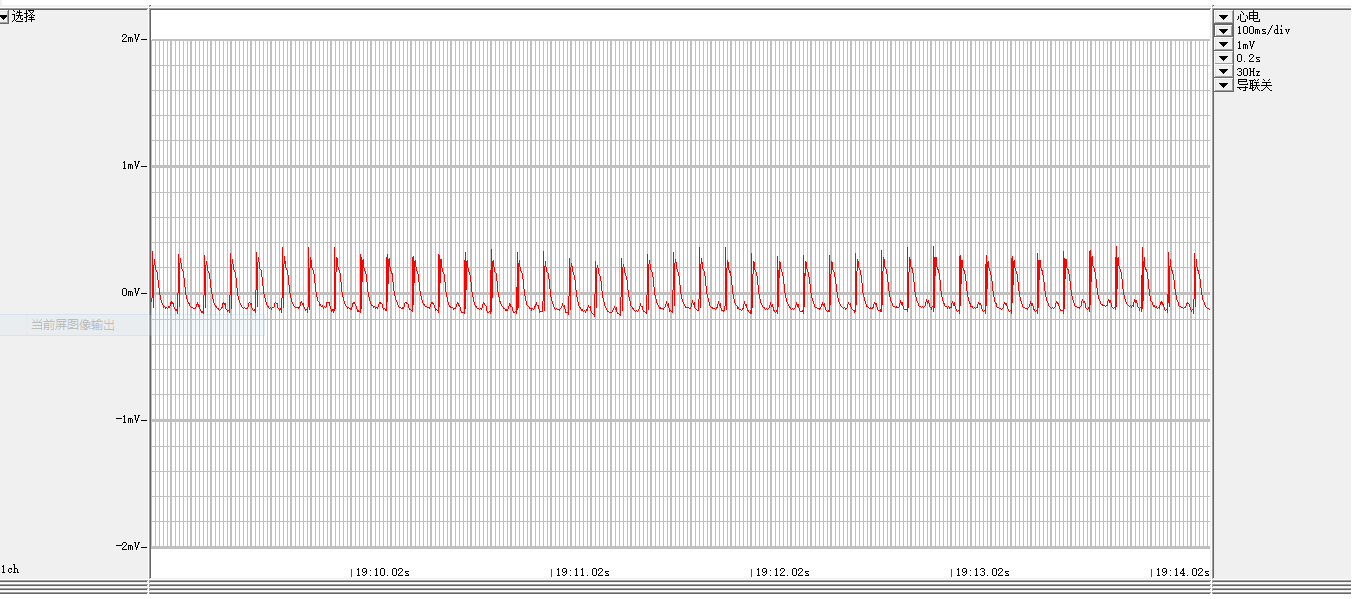
**

**Lidocaine**

**Pre-ligation-normal ECG**

**
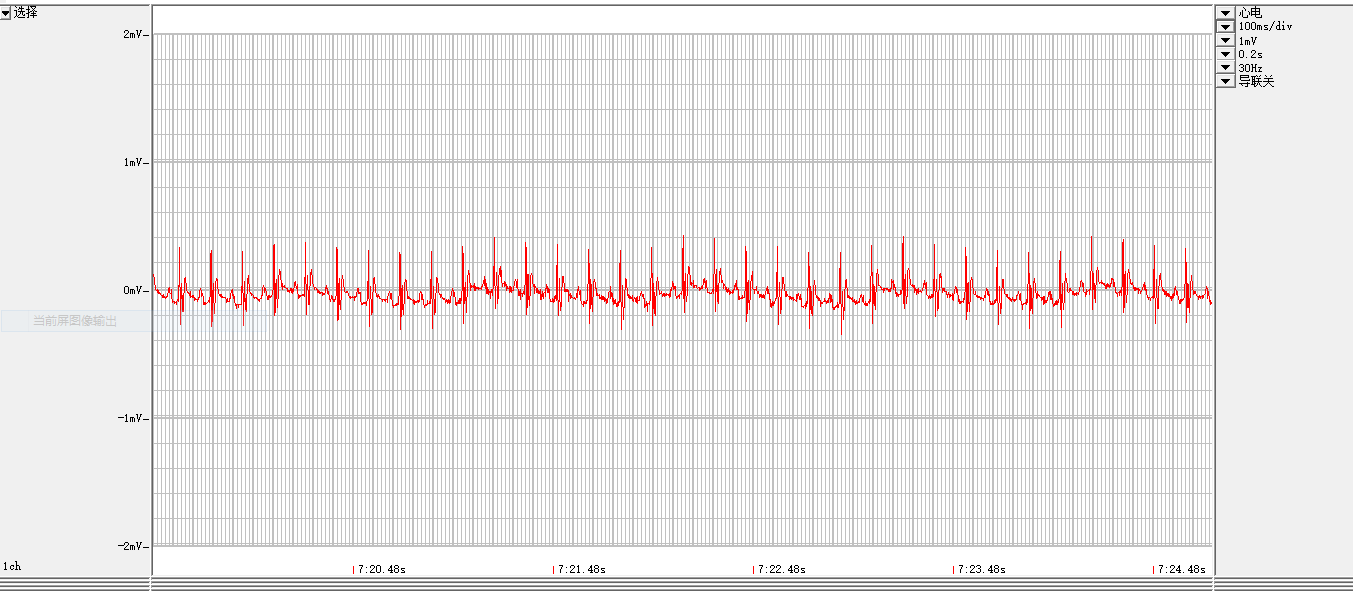
**

**Pre-ligation-Lidocaine, slightly slows the heart rhythm**

**
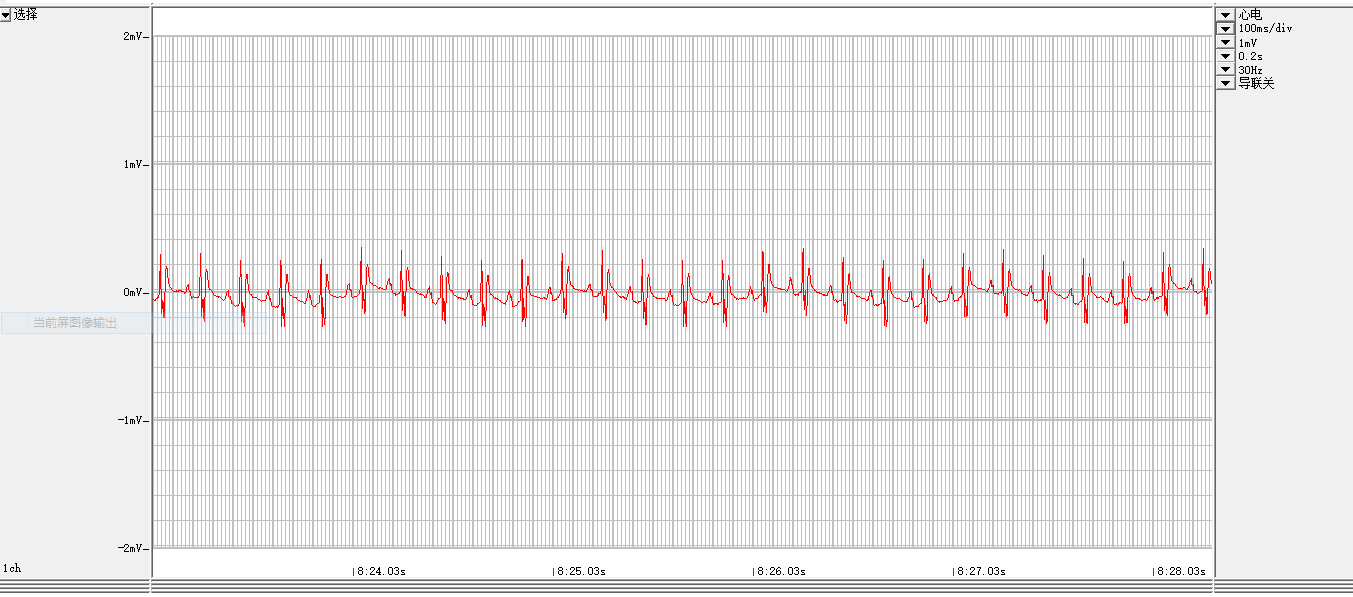
**

**Ligation-ST elevation**

**
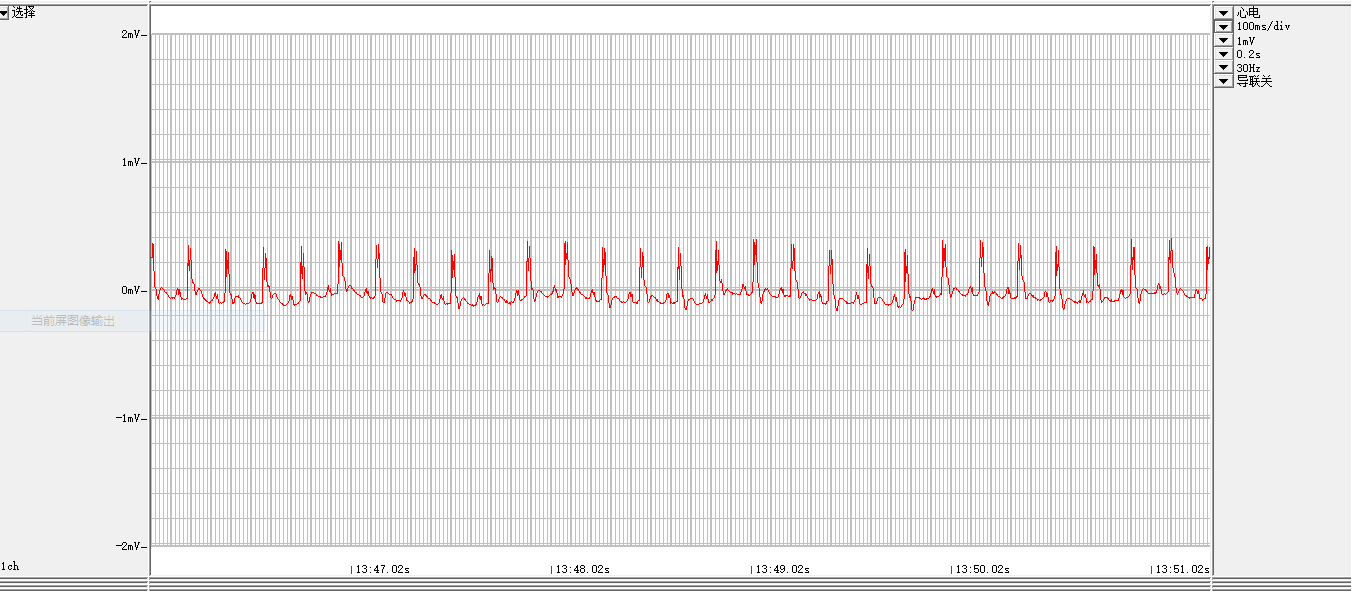
**

**Onset of arrhythmia, about 10 min post ligation**

**
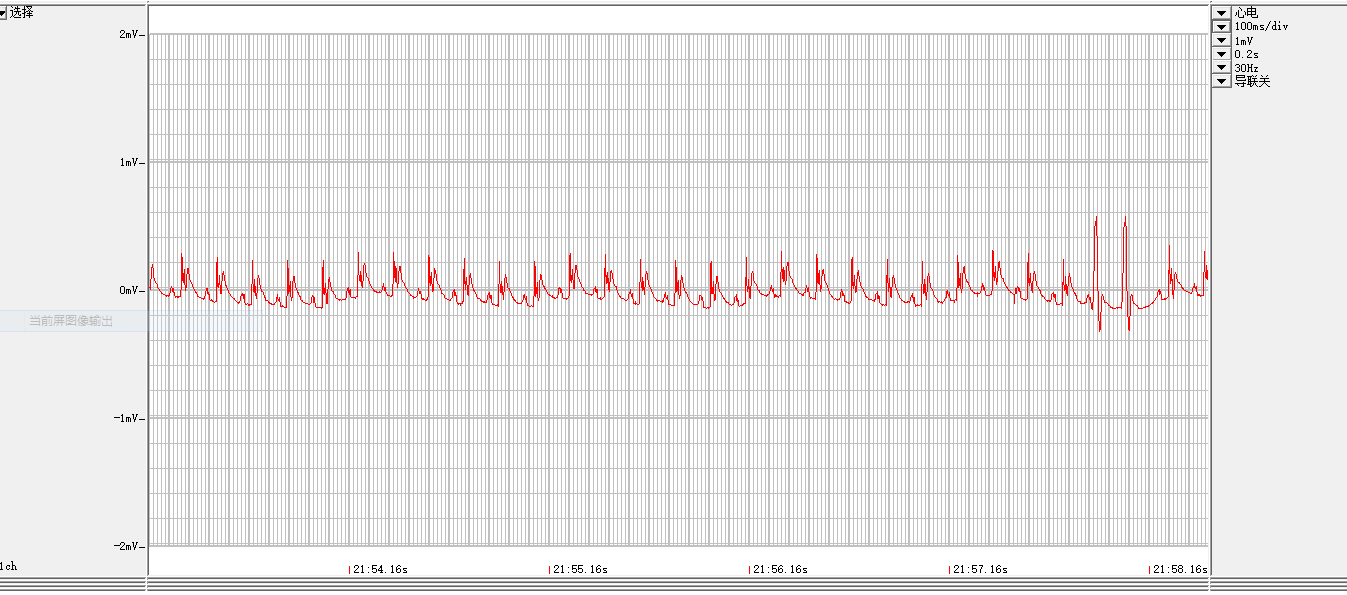
**

**Ligation-about 13min**

**
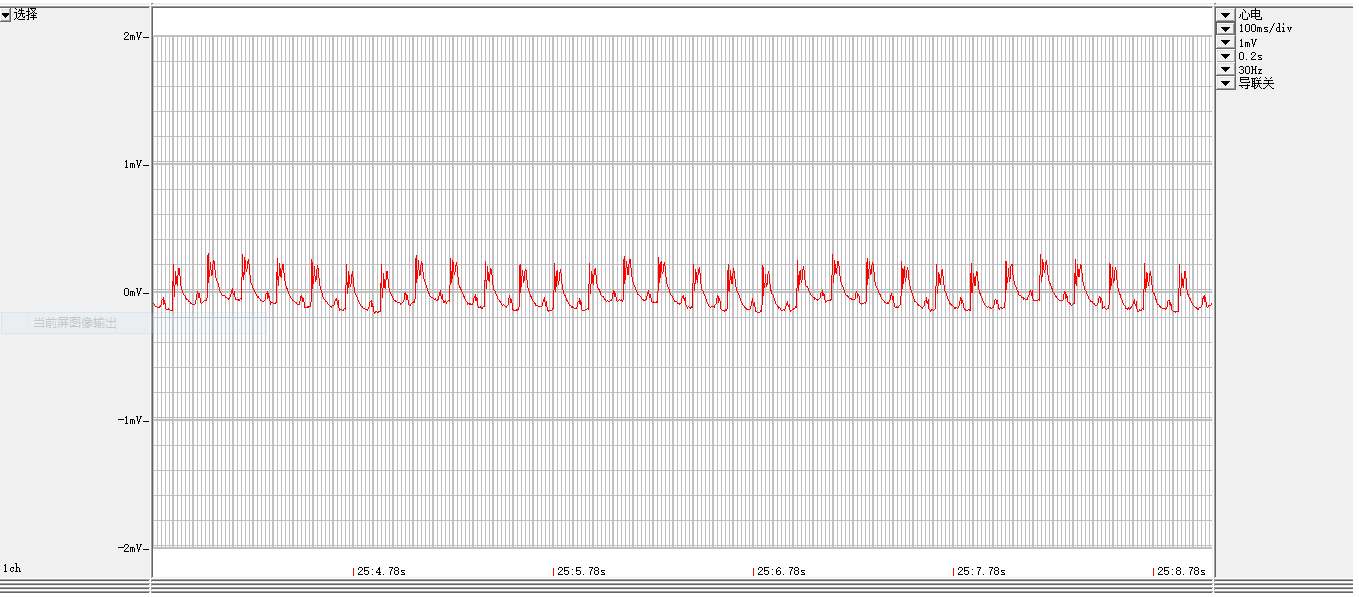
**

**2. Ex vivo - MI**

**Control**

**Pre-ligation- ECG**

**
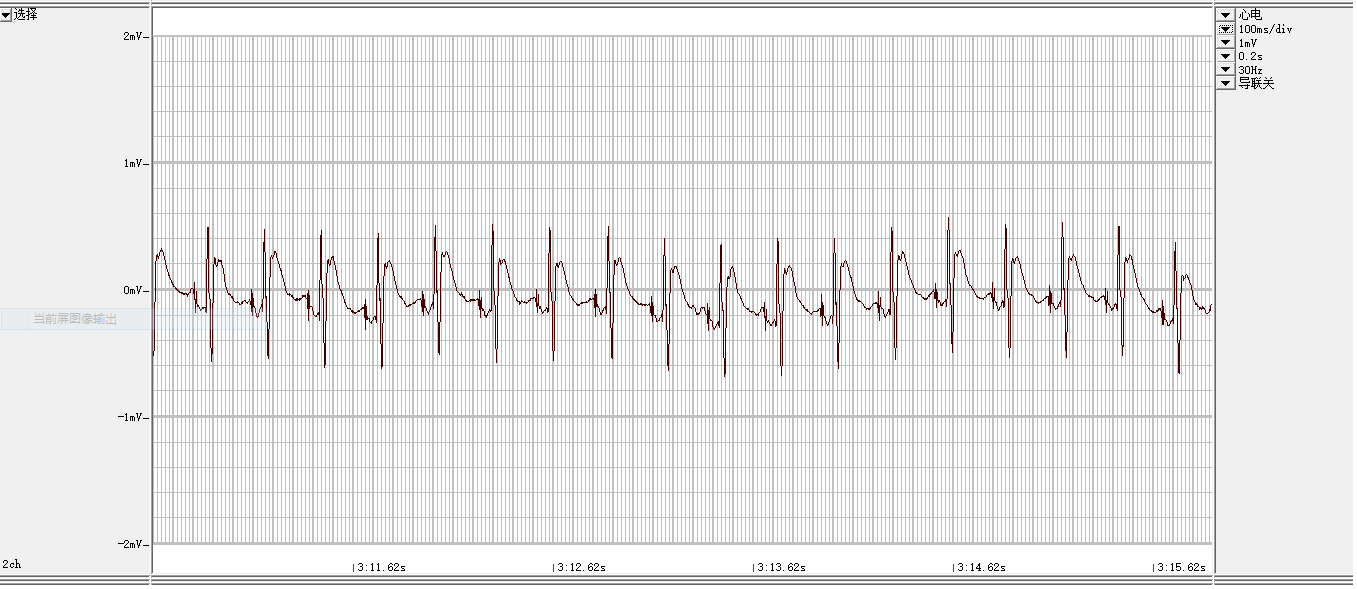
**

**Ligation-ST elevation**

**
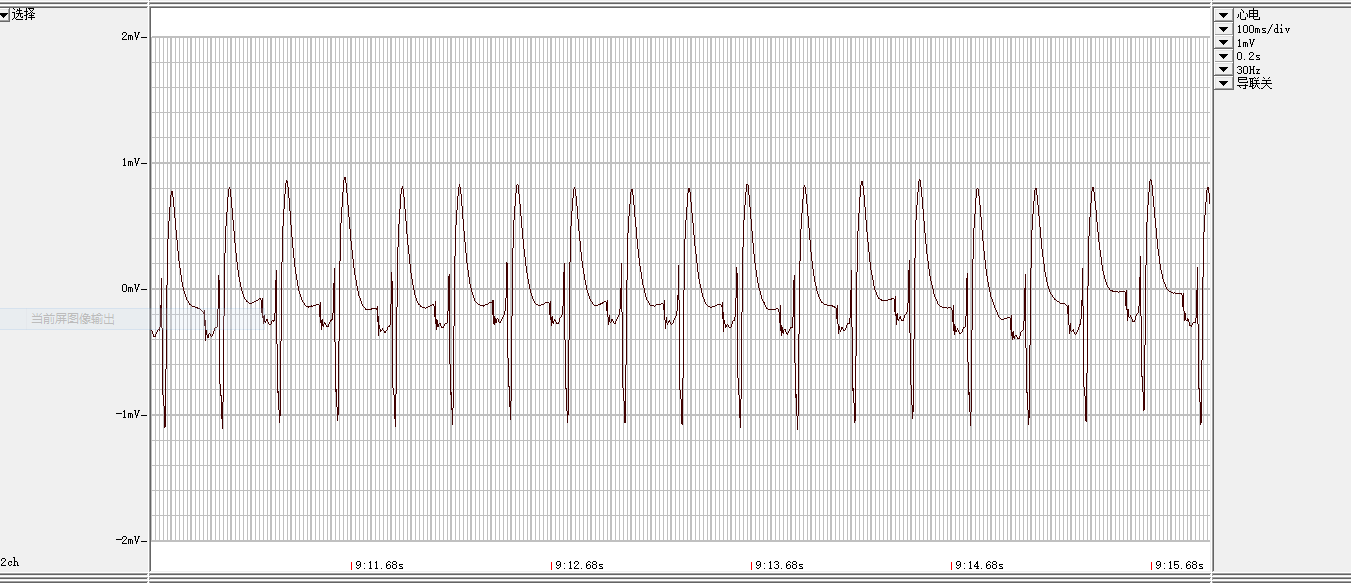
**

**Ligation**

**
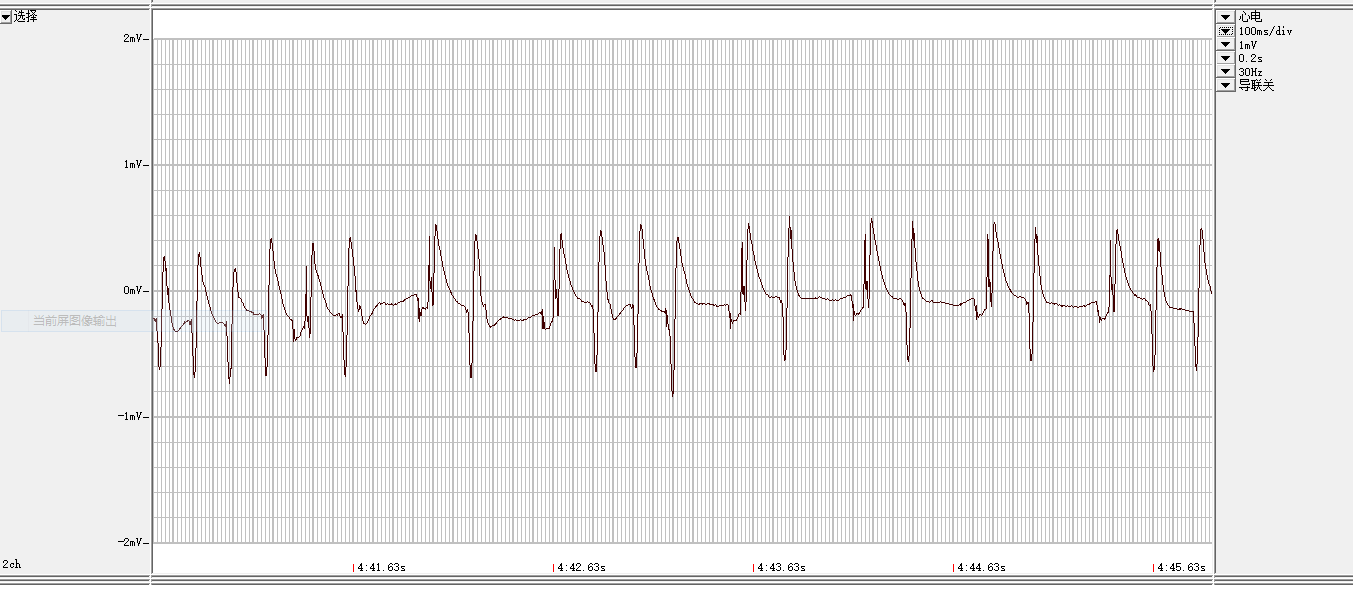
**

**
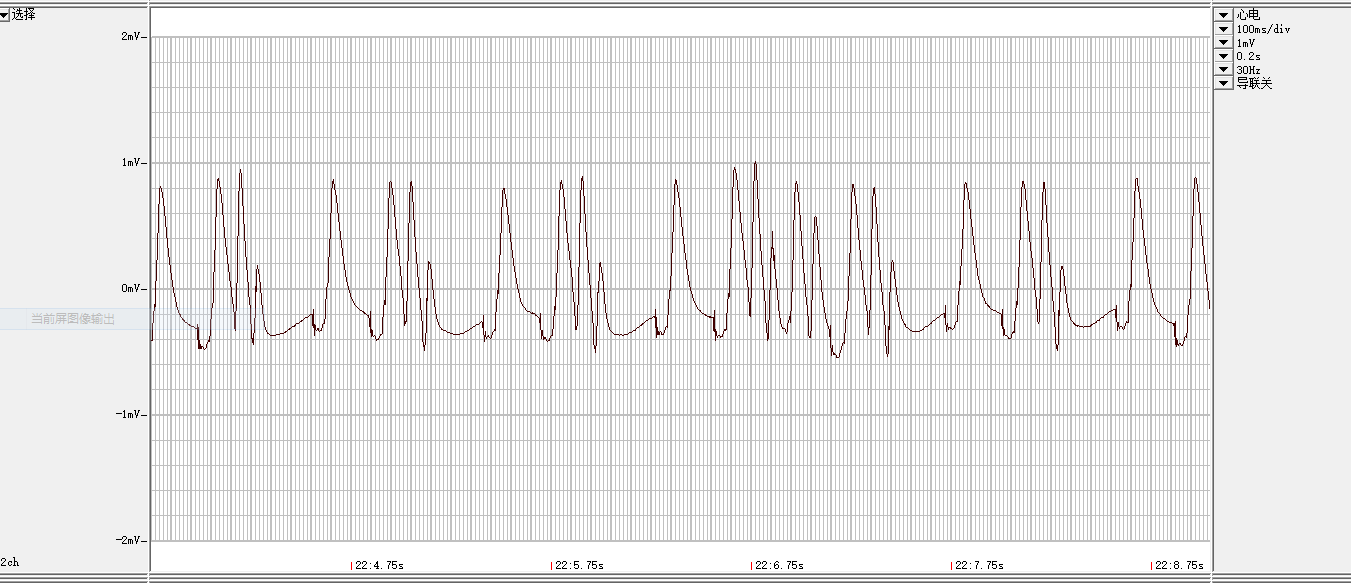
**

**
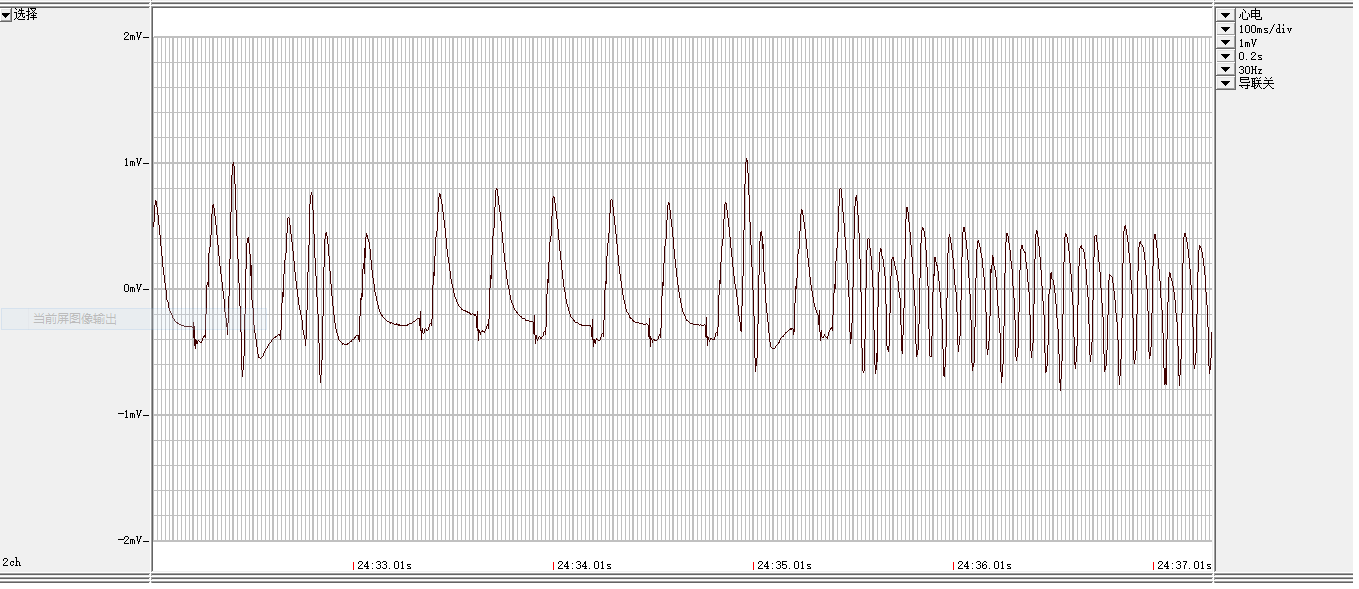
**

**Zacopride-1**

**Pre-ligation**

**
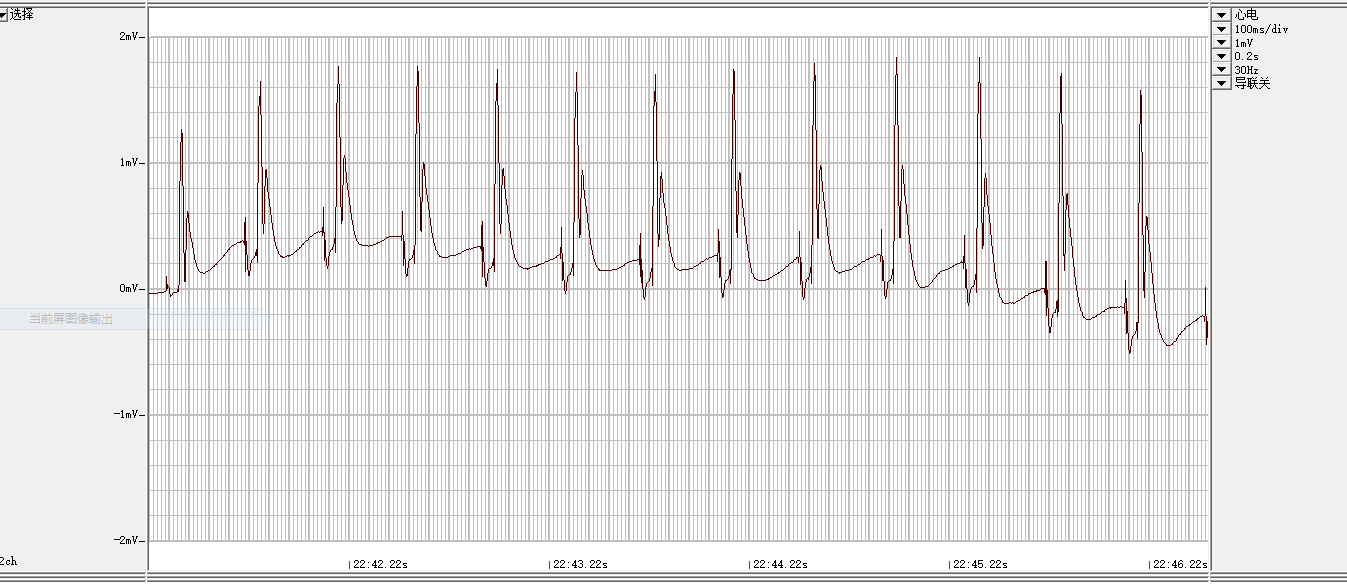
**

**Ligation-ST elevation**

**
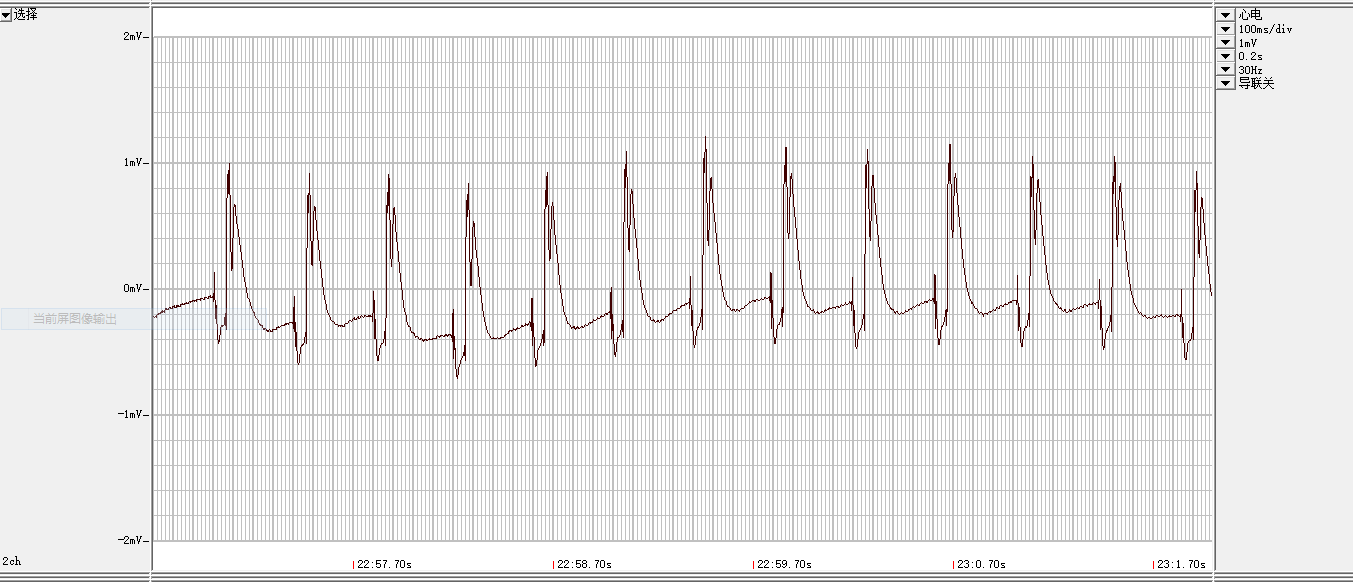
**

**Ligation**

**
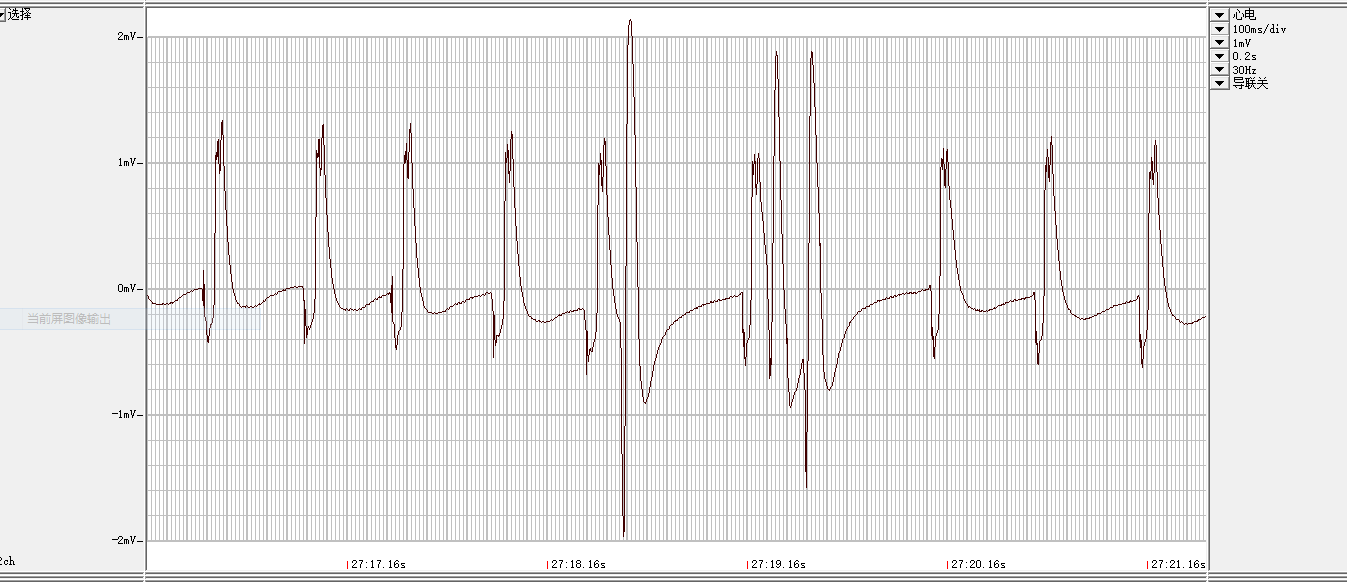
**

**
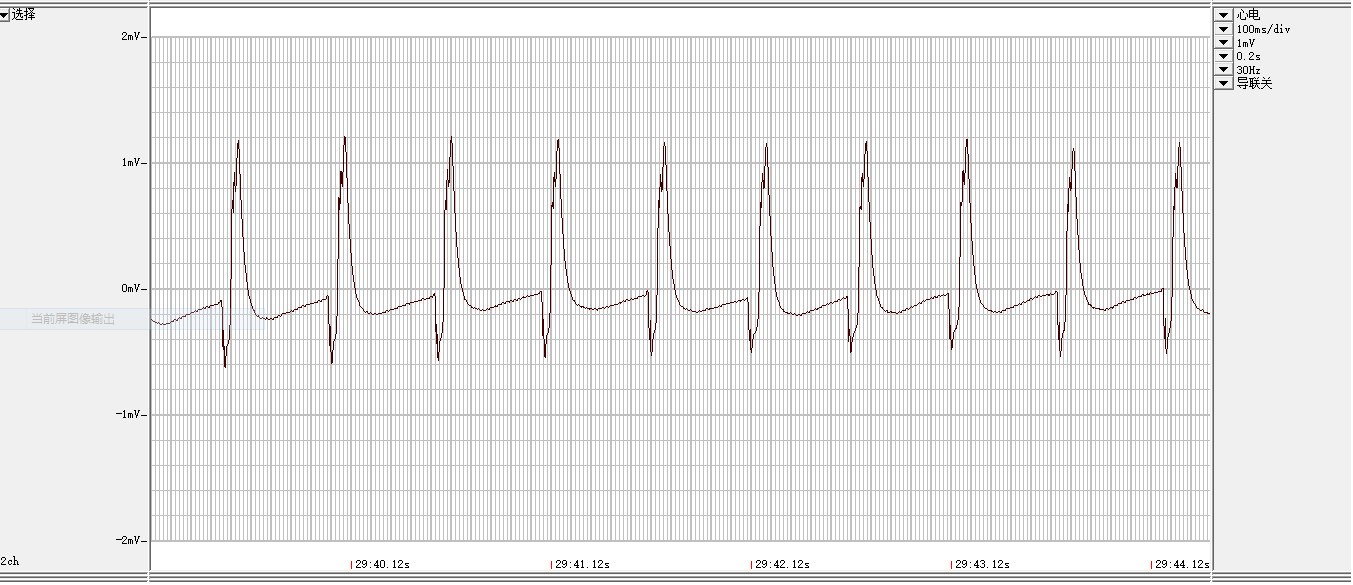
**

**
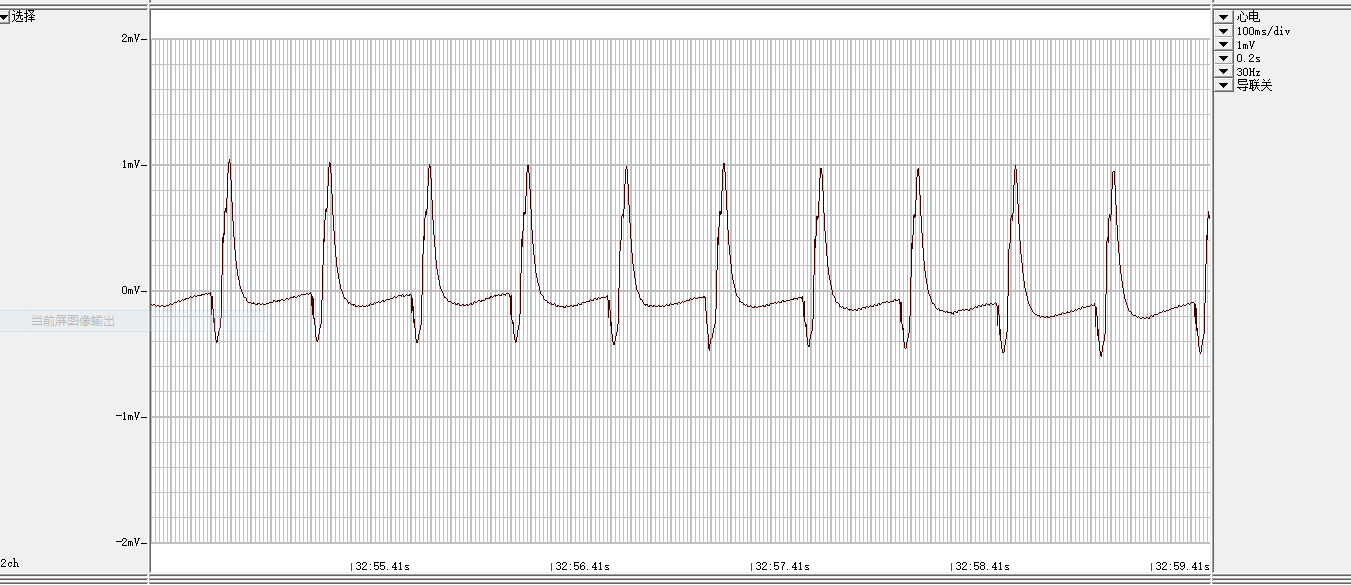
**

**Zacopride-2**

**Pre-ligation-normal ECG**


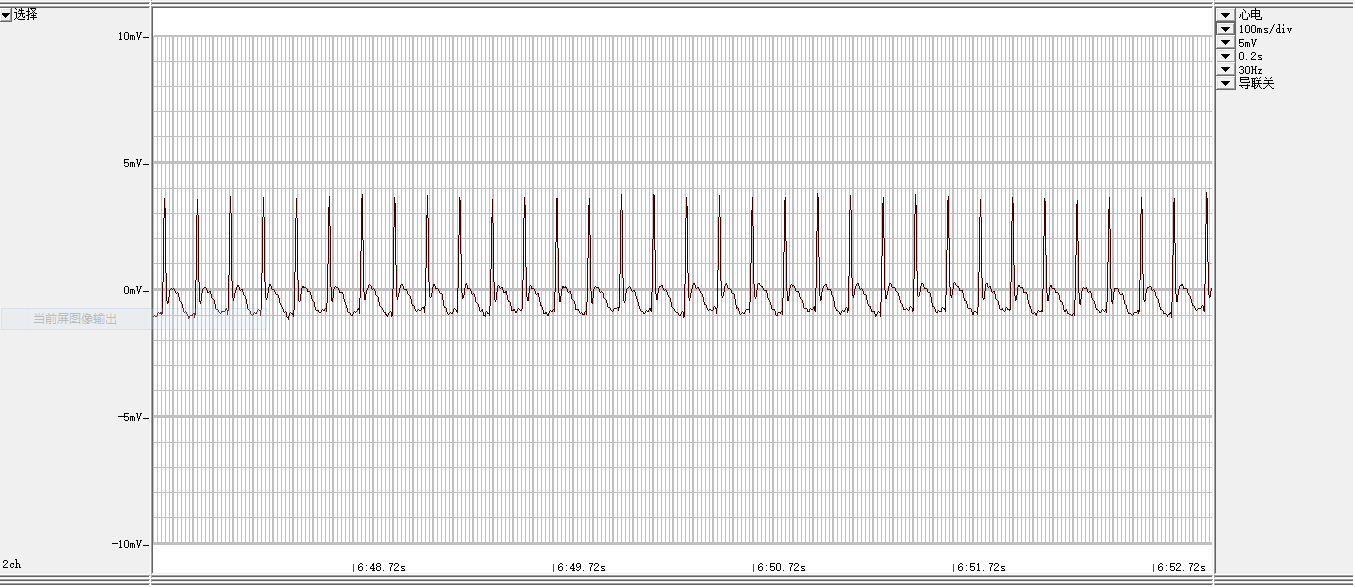


**Ligation-ST elevation**


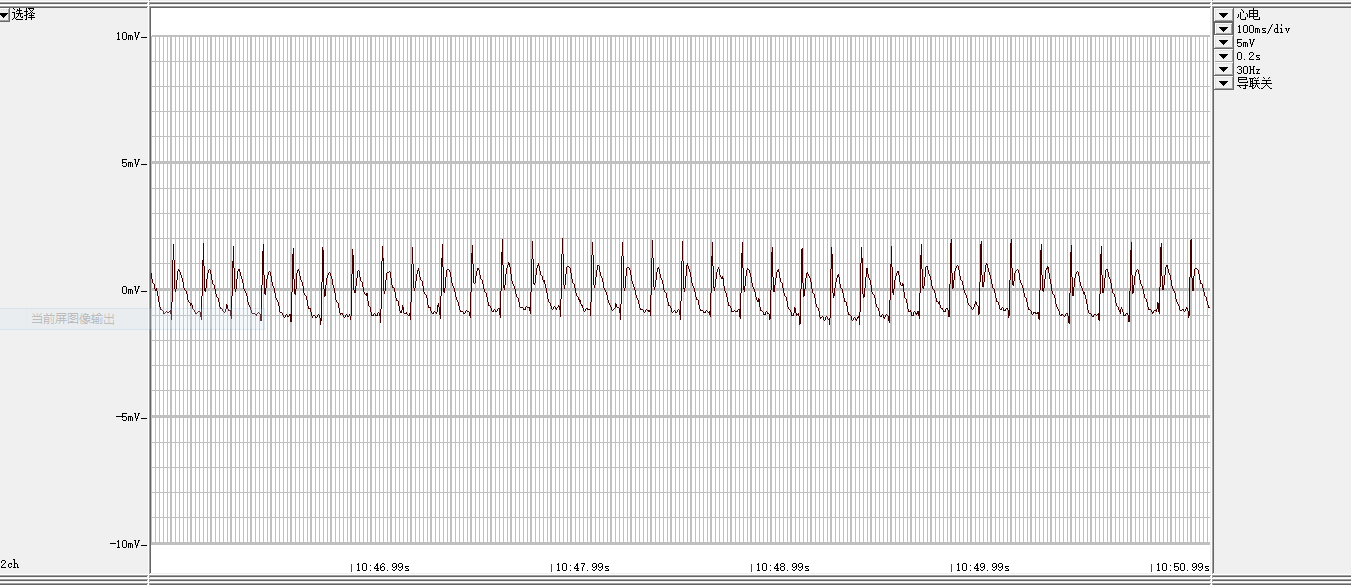


**Ligation**
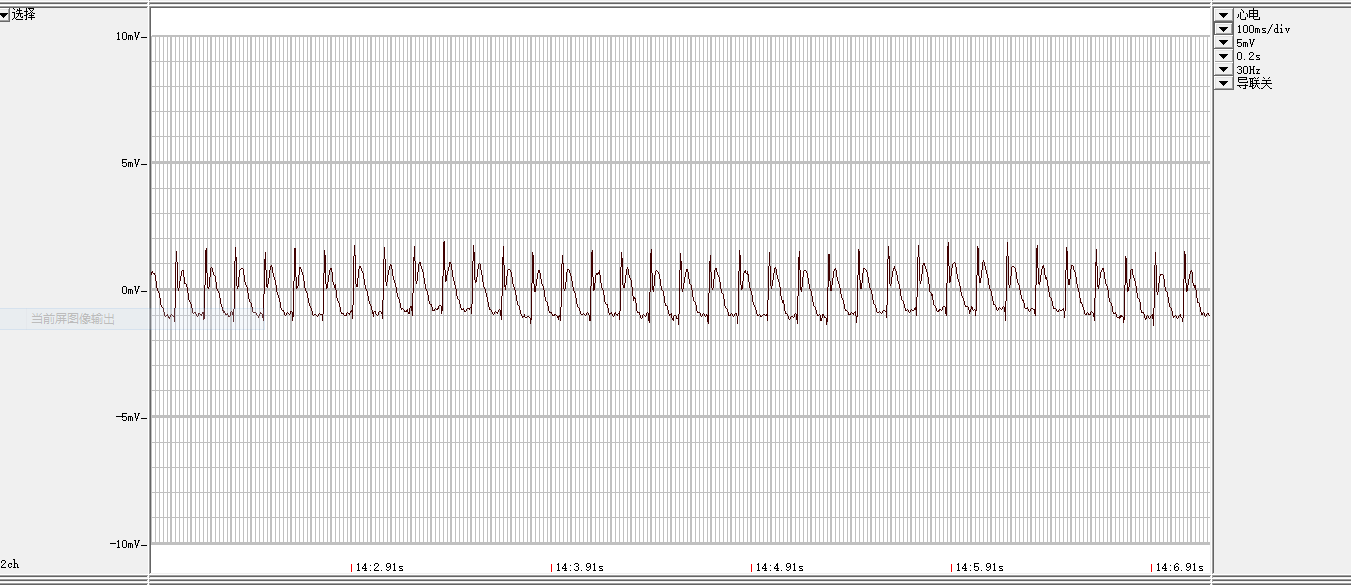

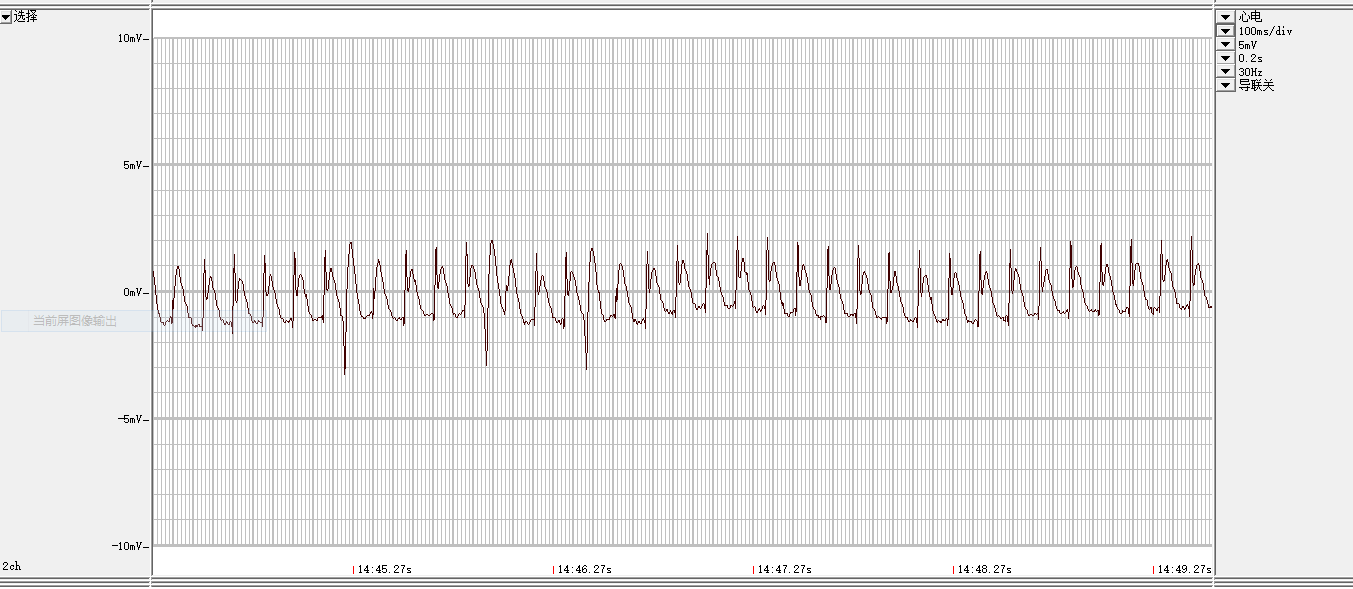


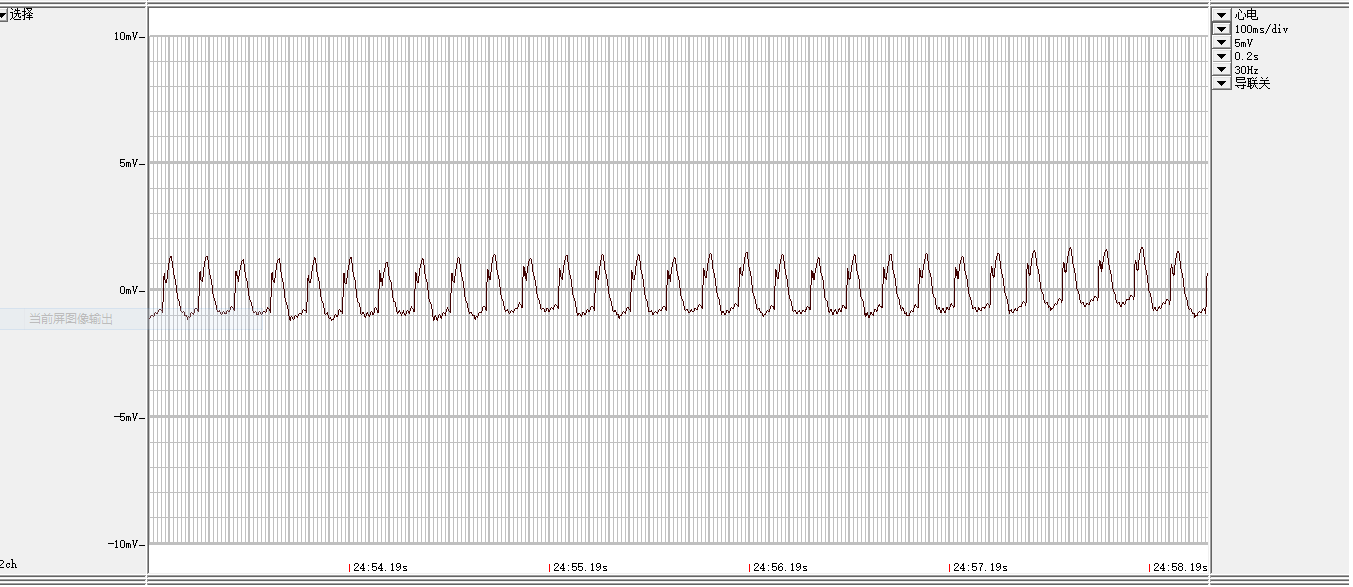


**Zacopride+BaCl_2_**

**Pre-ligation-normal ECG**

**_
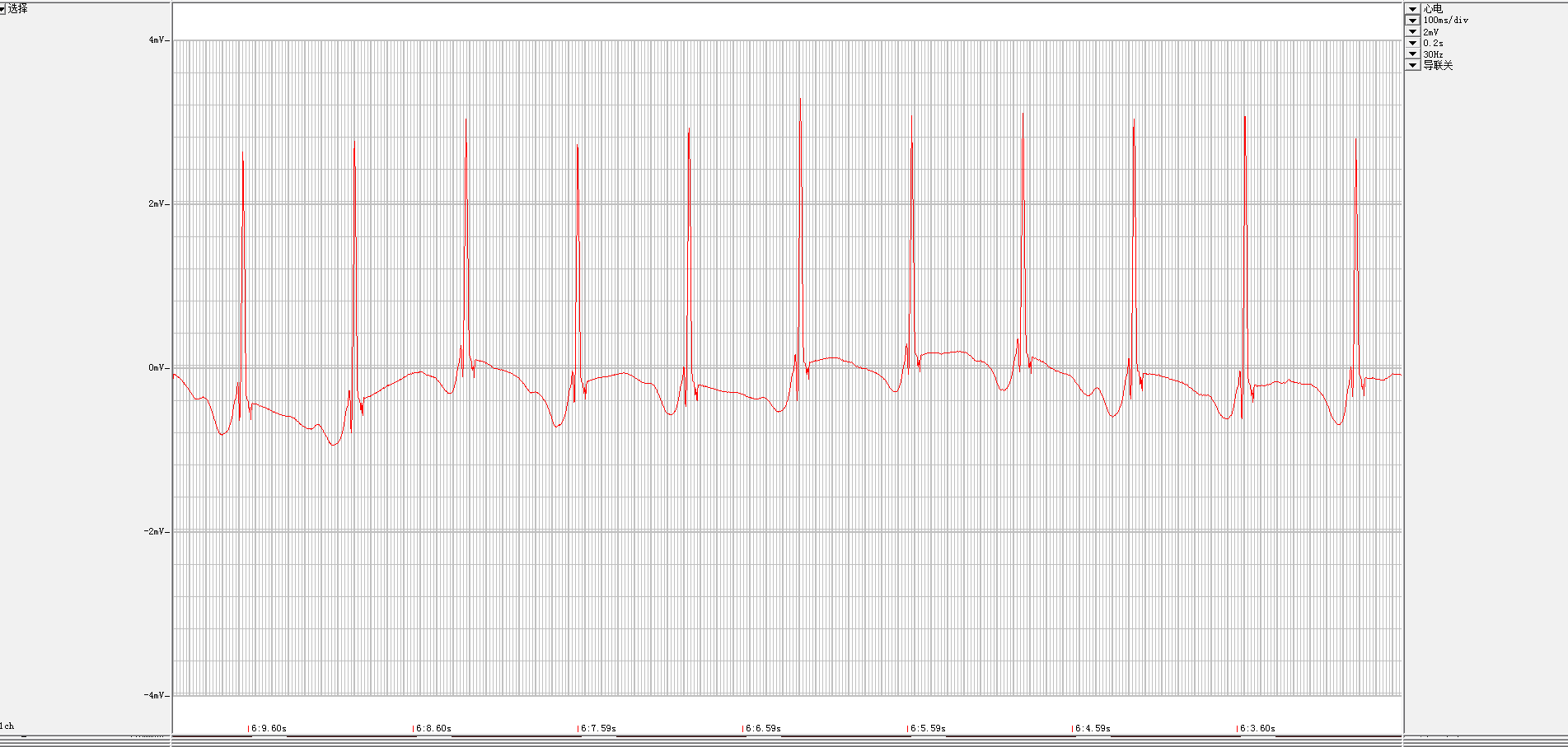
_**

**Pre-ligation- 1 μmol/L BaCl_2_**


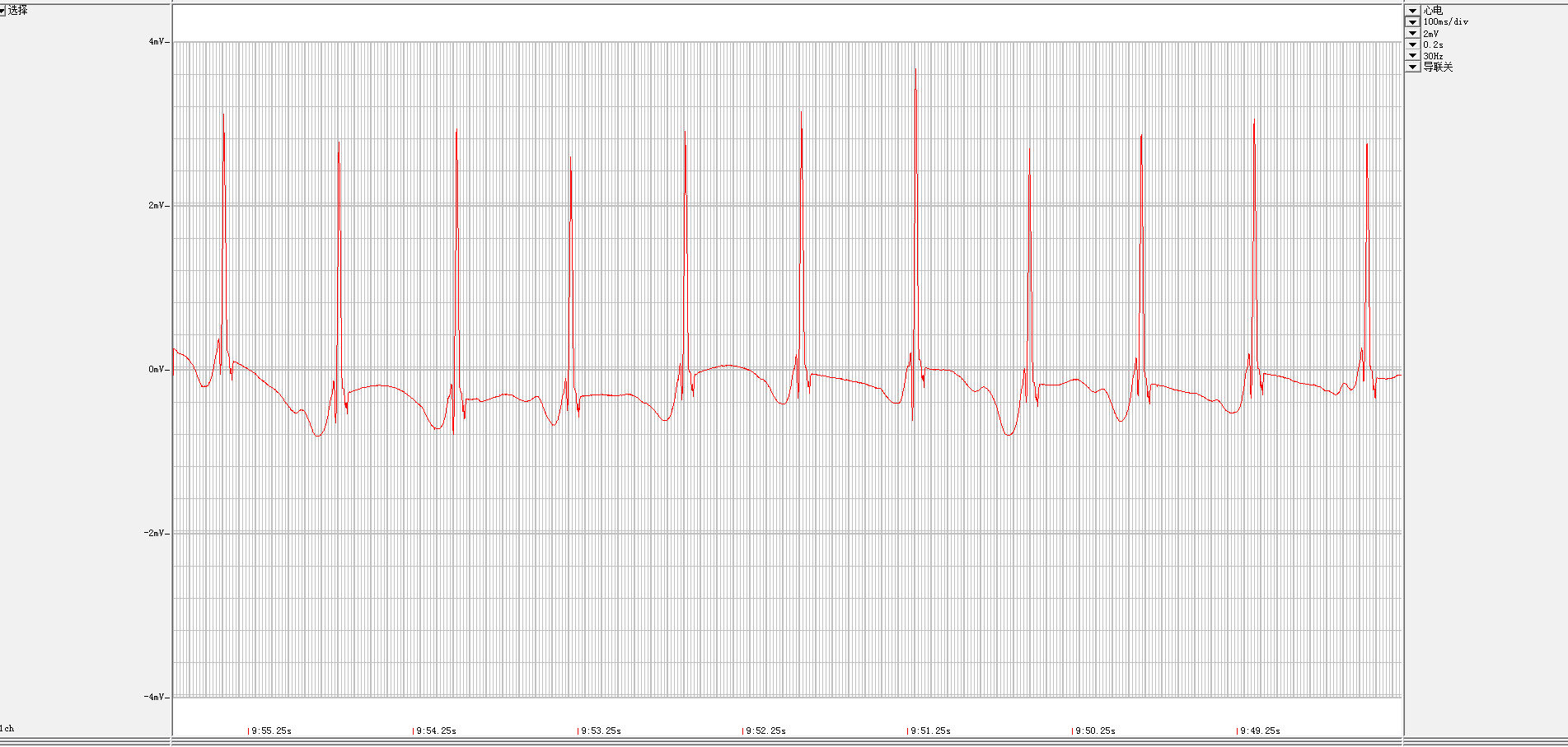


**ligation- 1 μmol/L BaCl_2_+1 μmol/L zacopride**


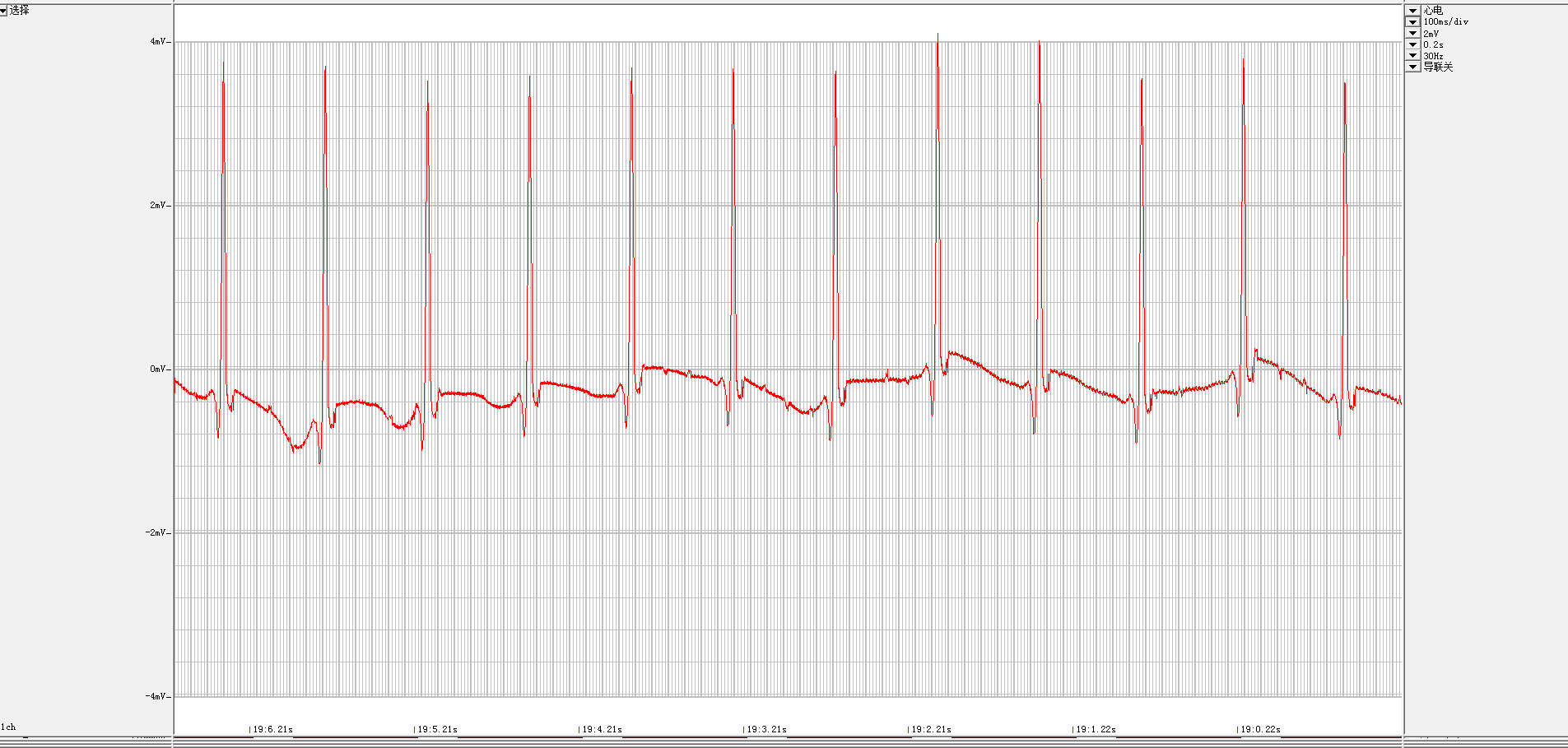


**ligation- 1 μmol/L BaCl_2_+1 μmol/L zacopride**

**
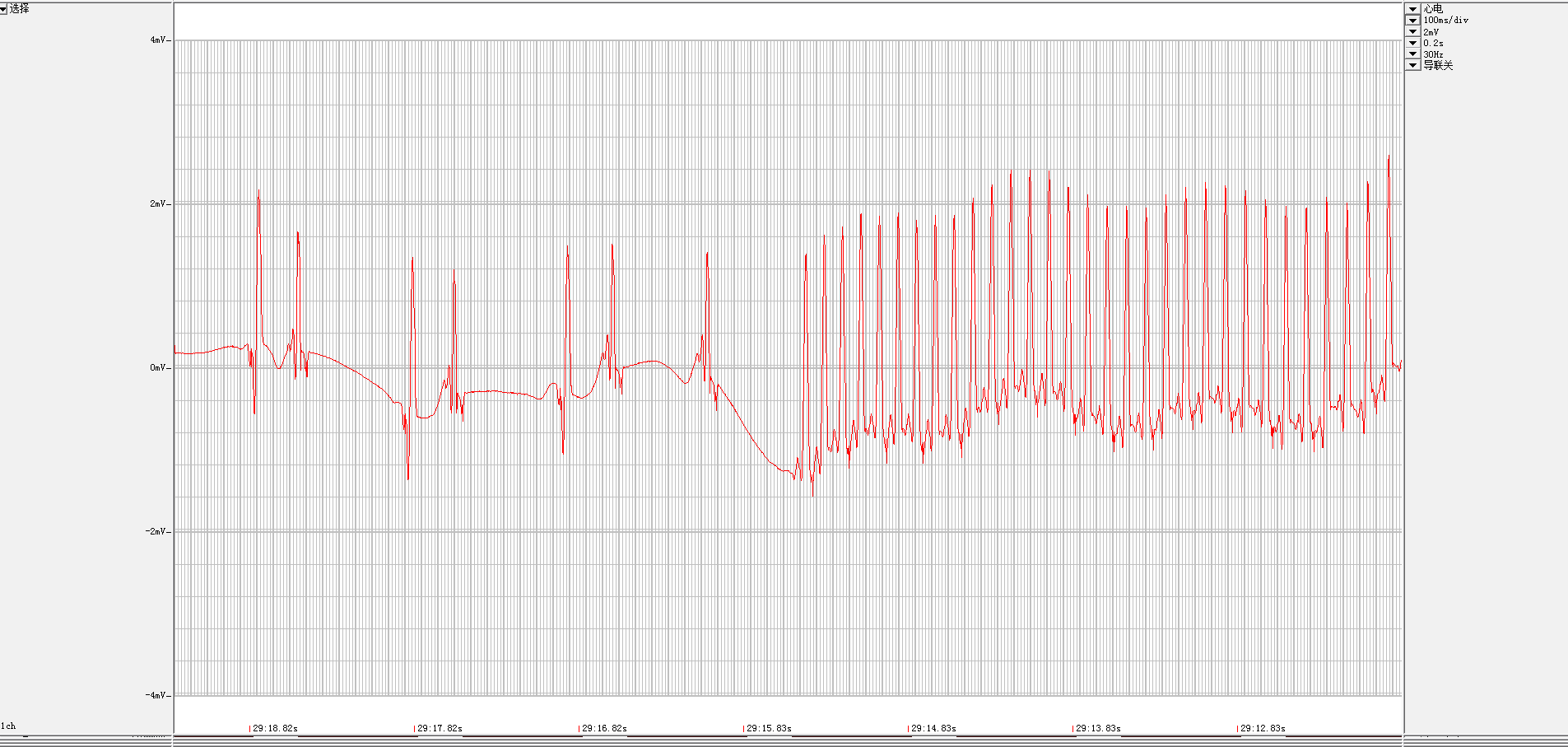
**

**
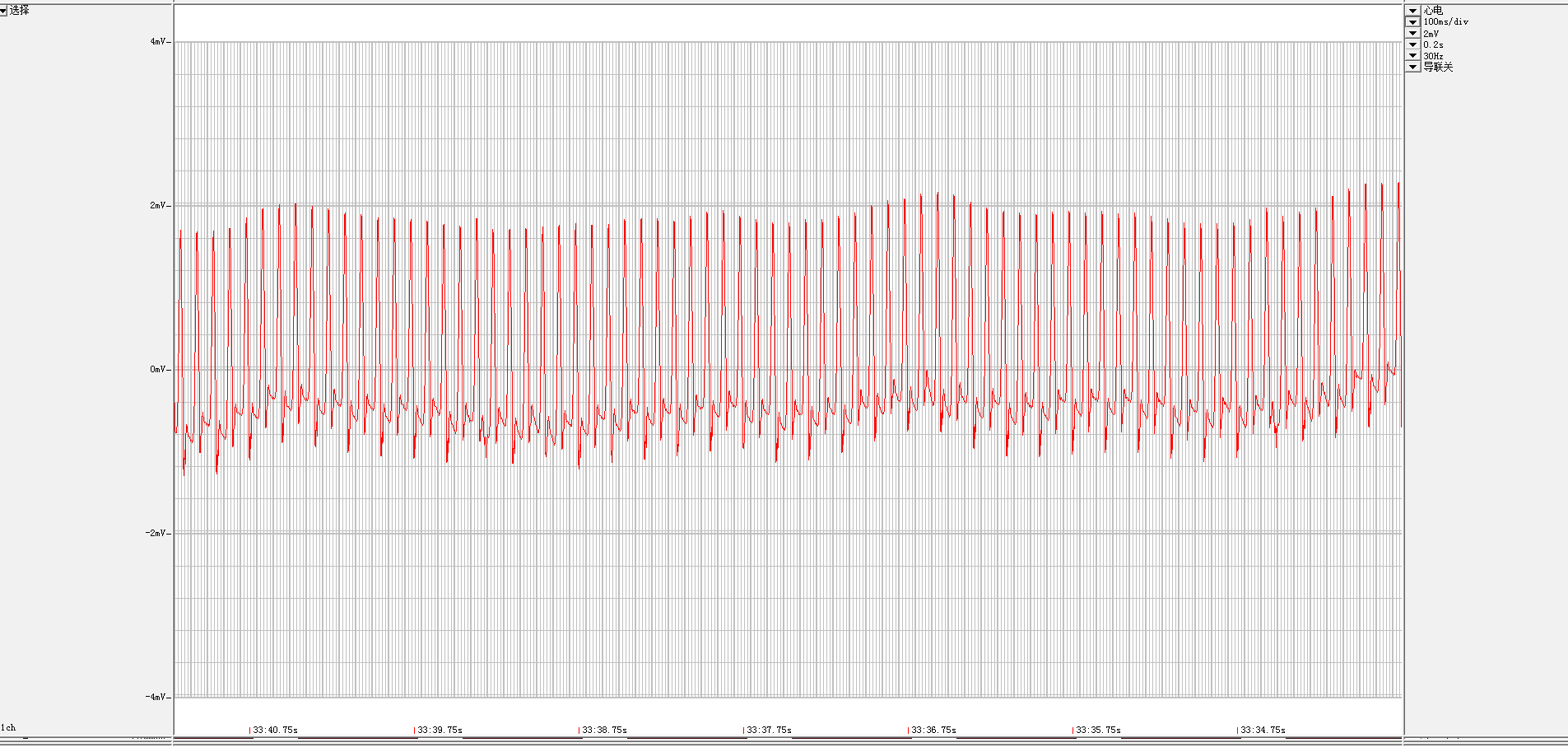
**

1. Patch clamp recording sweeps for I_K1_ in a rat ventricular myocyte

Zacopride restores hypoxia-induced *I*_K1_ decline. The increment of current was reversed by 1.0 µmol/L BaCl_2._

1. Patch clamp recording sweeps for Kir 2.1, Kir 2.2 and Kir 2.3 in CHO

Zacopride rescues the hypoxia-induced decline of *I*_Kir2.1_, but not I_Kir2.2_ and I_Kir2.3_, in CHO cells


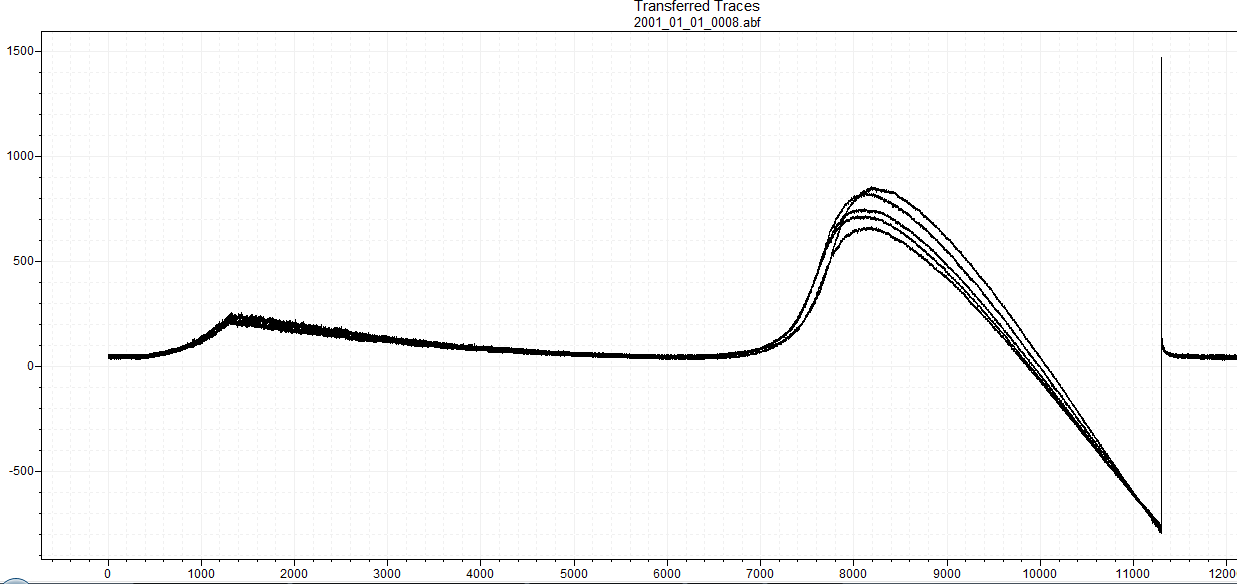


**Kir2.1**


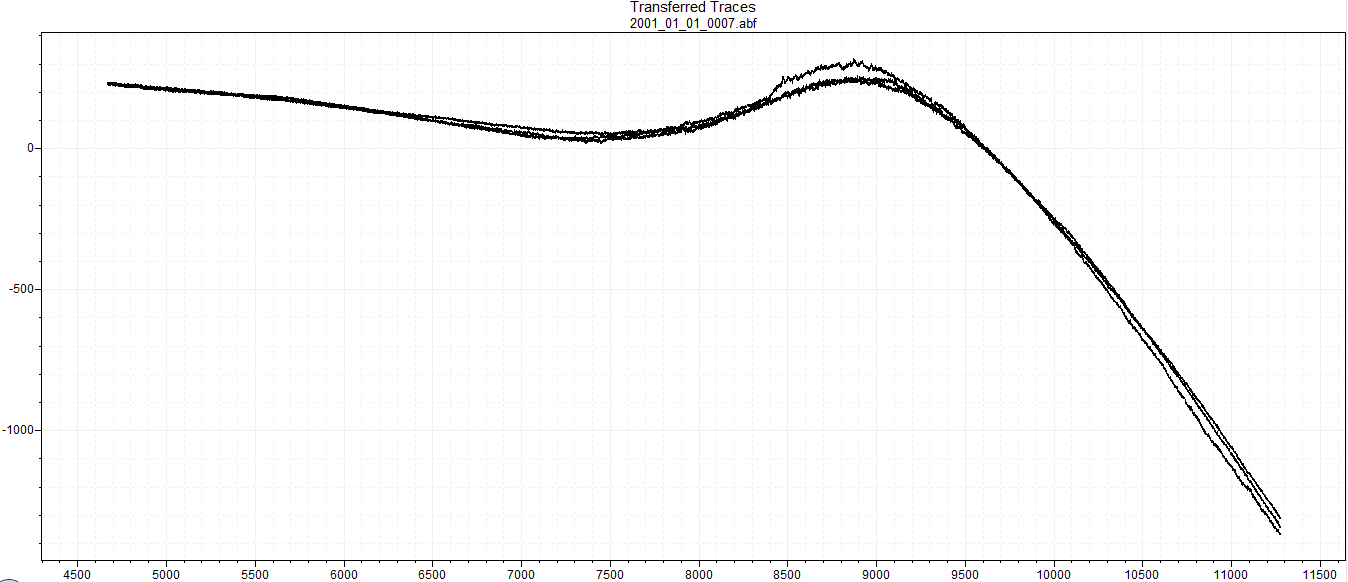


**Kir2.2**


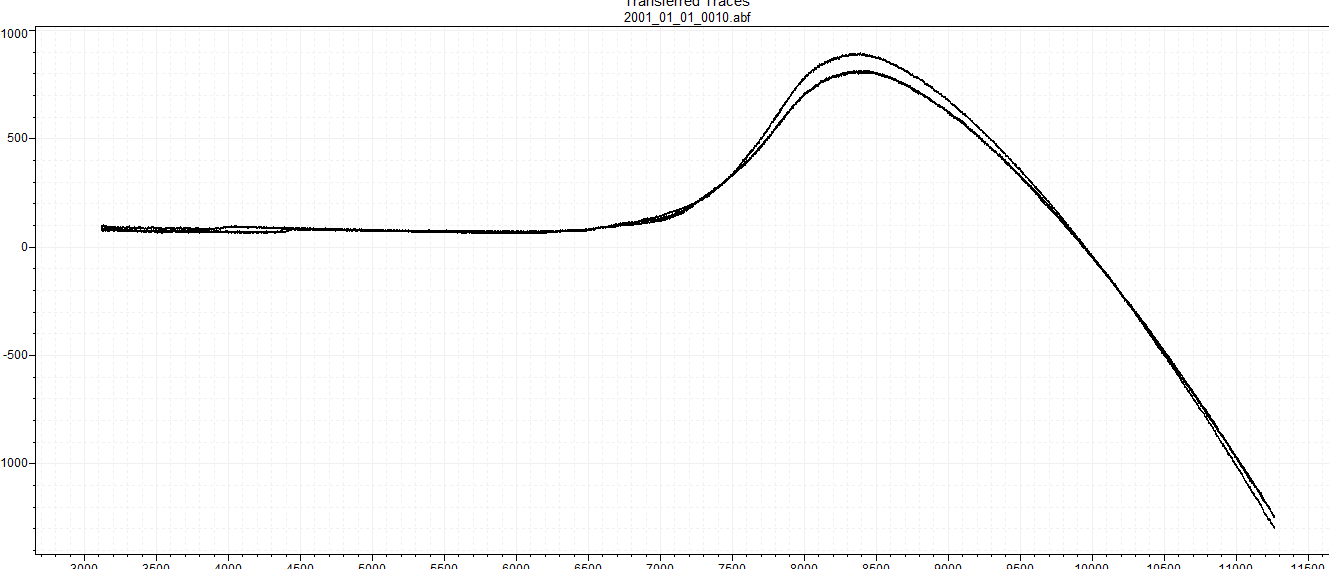


**Kir2.3**

1. **Zacopride restores hypoxia-induced RMP depolarization**


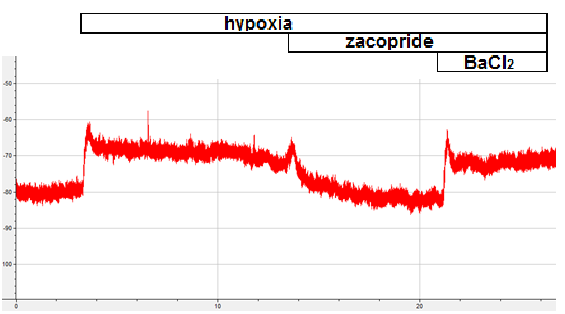


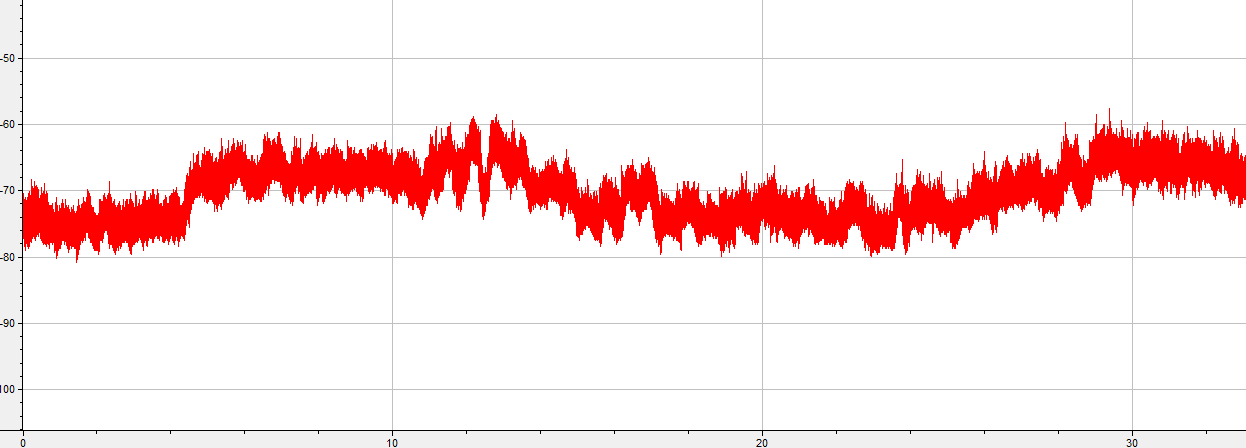

Supplement: S2 Dataset — (DOCX) [file pone.0177600.s002.docx]
